# The doublecortin-family kinase ZYG-8^DCLK1^ regulates microtubule dynamics and motor-driven forces to promote the stability of *C*. *elegans* acentrosomal spindles

**DOI:** 10.1371/journal.pgen.1011373

**Published:** 2024-09-03

**Authors:** Emily R. Czajkowski, Yuntong Zou, Nikita S. Divekar, Sarah M. Wignall

**Affiliations:** Department of Molecular Biosciences, Northwestern University, Evanston, Illinois, United States of America; Stowers Institute for Medical Research, UNITED STATES OF AMERICA

## Abstract

Although centrosomes help organize spindles in most cell types, oocytes of most species lack these structures. During acentrosomal spindle assembly in *C*. *elegans* oocytes, microtubule minus ends are sorted outwards away from the chromosomes where they form poles, but then these outward forces must be balanced to form a stable bipolar structure. Simultaneously, microtubule dynamics must be precisely controlled to maintain spindle length and organization. How forces and dynamics are tuned to create a stable bipolar structure is poorly understood. Here, we have gained insight into this question through studies of ZYG-8, a conserved doublecortin-family kinase; the mammalian homolog of this microtubule-associated protein is upregulated in many cancers and has been implicated in cell division, but the mechanisms by which it functions are poorly understood. We found that ZYG-8 depletion from oocytes resulted in overelongated spindles with pole and midspindle defects. Importantly, experiments with monopolar spindles revealed that ZYG-8 depletion led to excess outward forces within the spindle and suggested a potential role for this protein in regulating the force-generating motor BMK-1/kinesin-5. Further, we found that ZYG-8 is also required for proper microtubule dynamics within the oocyte spindle and that kinase activity is required for its function during both meiosis and mitosis. Altogether, our findings reveal new roles for ZYG-8 in oocytes and provide insights into how acentrosomal spindles are stabilized to promote faithful meiosis.

## Author summary

When a cell divides, a football-shaped spindle made up of dynamic microtubule polymers functions to partition chromosomes. Usually, structures called centrosomes organize microtubules at the two ends of the spindle. However, in female reproductive cells (“oocytes”) of many organisms, the spindle is formed without the help of centrosomes. In this study, we used the model organism *C*. *elegans* to understand how this type of “acentrosomal” spindle forms and maintains its shape. Specifically, we investigated a protein called ZYG-8 and we found that when this protein was removed from oocytes, the spindles became overelongated and their typical football shape was disrupted. Follow-up studies revealed that ZYG-8 plays two important functions that promote acentrosomal spindle assembly and stability in oocytes: 1) ZYG-8 helps control the dynamic properties of the microtubule polymers so that they can be properly arranged into the spindle structure and 2) ZYG-8 tunes forces within the spindle, to help the structure maintain it shape. Taken together, our findings have increased our understanding of how spindles form in the absence of centrosomes, shedding light on how female reproductive cells divide.

## Introduction

In most cell types, centrosomes function to nucleate and organize a microtubule-based spindle [1]. However, oocytes of most species lack centrosomes and yet are able to form a bipolar spindle [2,3]. In human oocytes, these acentrosomal spindles have been shown to be highly unstable; after bipolarity is achieved, the poles often split apart, and this instability leads to a high incidence of chromosome segregation errors [4]. The mechanisms by which oocyte spindles are formed and stabilized are thus important to understand.

In recent years, *C*. *elegans* has emerged as a powerful model to dissect mechanisms that promote acentrosomal spindle assembly and maintenance, since rapid depletion methods have been developed that enable the removal of proteins from oocytes within minutes [5,6]. These methods have made it possible to either deplete proteins prior to spindle formation, to probe for roles in spindle assembly, or to remove proteins from pre-formed spindles and then assess effects on the stability of the structure [7–9].

In *C*. *elegans* oocytes, assembly of the spindle begins with nuclear envelope breakdown. Microtubules initially nucleate and form a cage-like structure around the meiotic chromosomes. Subsequently, microtubule minus ends are sorted outwards to form multiple poles that eventually coalesce into two distinct poles [10–12]. Thus, to generate a stable spindle structure, forces must be produced during spindle assembly to sort microtubules outwards, and then these forces must be balanced after the spindle forms to maintain proper spindle size. KLP-18/kinesin-12, a plus-end directed microtubule motor, sorts microtubule minus ends outwards to establish bipolarity; depletion of KLP-18 results in a monopolar spindle in which microtubule minus ends coalesce into a single pole at the center with plus ends radiating outwards [10,13]. KLP-18 is also required to maintain spindle bipolarity; if KLP-18 is inactivated after the bipolar spindle has already formed, the two poles converge together to create a monopolar spindle [14].

A recent study demonstrated that in addition to KLP-18, another plus end directed motor, BMK-1/kinesin-5, also provides outward force on the spindle. Although BMK-1 depletion does not cause defects on its own since KLP-18 provides the primary outward force [15,13], a role for BMK-1 was revealed in experimental conditions where KLP-18 was depleted [8]. Interestingly, this study also demonstrated that dynein provides an inward force on the spindle; acute dynein depletion caused the spindle to elongate and the poles to splay. Thus, a balance of motor-generated outward and inward forces is essential to form and maintain the acentrosomal spindle in *C*. *elegans* oocytes.

In addition to motors, other factors are also necessary for spindle stability. Removal of the microtubule polymerase ZYG-9 from pre-formed spindles causes microtubules in the center of the spindle to splay and poles to split, suggesting that regulation of microtubule dynamics is essential for the spindle to maintain its structure [9]. Similarly, it has been shown that control of microtubule length and number are essential for proper spindle assembly. Depletion of the microtubule depolymerase MCAK results in the formation of disorganized spindles with longer microtubules [11,12,16,17]. Moreover, mutating the microtubule severing enzyme katanin entirely prevents spindle assembly [18–20]; this enzyme converts long microtubules into shorter fragments, thus increasing polymer number to promote spindle assembly [21]. Therefore, precise tuning of microtubule number, length, and dynamics is essential for the spindle to properly form and maintain its structure.

Although it is known that force balance and microtubule dynamics must be precisely controlled in the acentrosomal spindle, the mechanisms by which this control is achieved are not well understood. Here, we provide insight into these mechanisms through analysis of ZYG-8, a conserved microtubule-associated protein. ZYG-8 contains a microtubule-binding doublecortin domain and a kinase domain [22]. Previous studies using a series of temperature-sensitive *zyg-8* mutants demonstrated that ZYG-8 is required for proper spindle positioning in *C*. *elegans* mitotically dividing embryos [22,23]. Moreover, analysis of astral microtubule dynamics demonstrated that ZYG-8 depletion led to reduced growth rates and increased nucleation rates, demonstrating that ZYG-8 modulates microtubule dynamics in mitosis [24]. In oocytes, ZYG-8 is required for anaphase B spindle elongation [25] and a recent study demonstrated that it excludes the microtubule crosslinker SPD-1^PRC1^ from the anaphase spindle, thus regulating spindle structure during chromosome segregation [26]. While this study also noted that there were metaphase spindle defects following ZYG-8 depletion, the question of how ZYG-8 facilitates proper spindle organization was not explored.

Here, we show that ZYG-8 is required for both the assembly and stability of the oocyte spindle. Further, we demonstrate that ZYG-8’s kinase activity is required for its functions in both mitosis and meiosis, and we reveal important roles for this protein in modulating both microtubule dynamics and motor-driven forces within the oocyte spindle. Together, this work reveals new functions for ZYG-8 during meiosis and sheds light on how the proper structure of the oocyte spindle is achieved and maintained.

## Results

### Temperature sensitive *zyg-8* mutants have oocyte spindle defects

**Fig 1:**
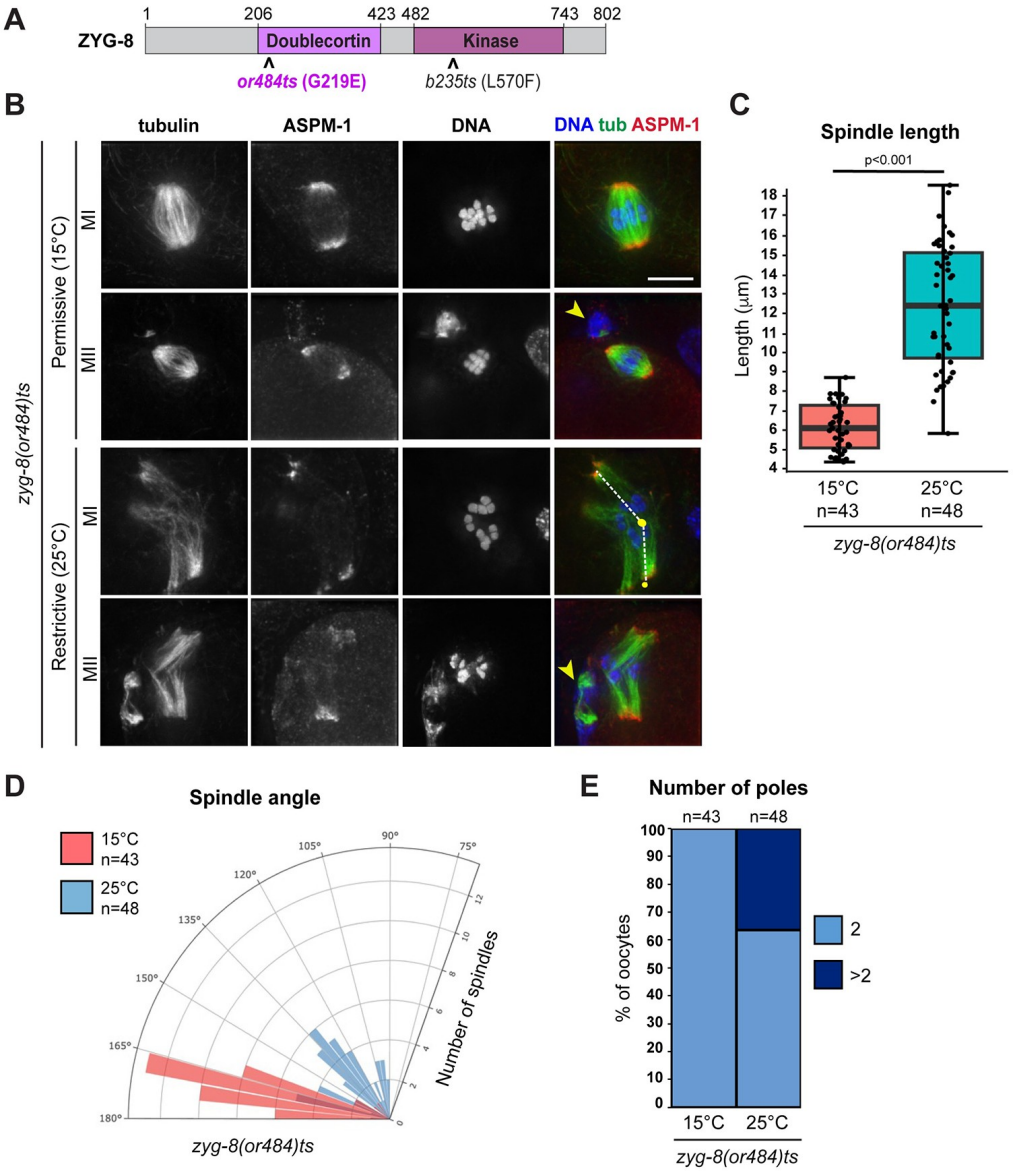
A *zyg-8* temperature sensitive mutant displays defects in oocyte spindle morphology at the restrictive temperature. (A) ZYG-8 schematic, highlighting the microtubule binding doublecortin domain, the kinase domain, and the location of the *or484* and *b235* temperature sensitive mutations. (B) Immunofluorescence images of *zyg-8(or484)* oocytes at either the permissive (15°C) or restrictive (25°C) temperature. Shown are tubulin (green), DNA (blue), and ASPM-1 (red). Similar defects were observed in both Meiosis I and Meiosis II; yellow arrowheads denote polar bodies. Lines drawn on the merged image in the third row represent how spindle angles were measured for the quantification shown in panel D. (C-E) Quantification of spindle length, spindle angle, and number of ASPM-1-marked poles. After incubation at the restrictive temperature, oocyte spindles were on average longer (p<0.001), more bent (p<0.0001), and some had additional ASPM-1-marked poles. Scale bar = 5μm.

To begin investigating the function of *C*. *elegans* ZYG-8 in acentrosomal spindle assembly, we utilized two different *zyg-8* temperature sensitive mutant strains, one with a mutation within the microtubule-binding Doublecortin domain, *zyg-8(or484)*, and the other with a mutation within the kinase domain, *zyg-8(b235)* (Figs 1A **and** S1A). These mutants have been previously characterized and both display mitotic defects at the restrictive temperature of 25°C [22,23]. Following incubation of these strains at this temperature, we found that oocyte spindles displayed various morphological defects (Figs 1B–1E **and** S1B–S1E); these defects were observed in both Meiosis I and Meiosis II (Fig 1B), so all quantifications represent a mix of these two stages. First, staining with the microtubule minus end marker ASPM-1 revealed pole defects; while spindles at the permissive temperature had two ASPM-1 clusters, an increased number of spindles at the restrictive temperature had 3 or more clusters, reflecting that the spindles were multipolar and/or had fragmented poles (18/48 *or484* spindles and 24/62 *b235* spindles; Figs 1E **and** S1E). Spindles at 25°C were also longer than control spindles (Figs 1C **and** S1C), and many spindles appeared to be bent. To quantify this phenotype, we used ASPM-1 staining to define the center point of each pole. We then drew lines connecting each pole to the middle of the spindle, defined by the center of the DNA staining, and measured the angle between the two poles (Fig 1B schematic); a perfectly straight spindle would therefore measure 180 degrees. Using this measurement, we found that spindles at the restrictive temperature were on average significantly more bent than at the permissive temperature (Figs 1D **and** S1D). In contrast, the spindle lengths and angles of the two *zyg-8ts* strains at the permissive temperature were not significantly different from wild-type spindles (S1F and S1G Fig). Together, these results suggest that ZYG-8 is required for proper spindle assembly in oocytes.

### ZYG-8 localizes diffusely across the meiotic spindle and is required for proper spindle assembly

**Fig 2:**
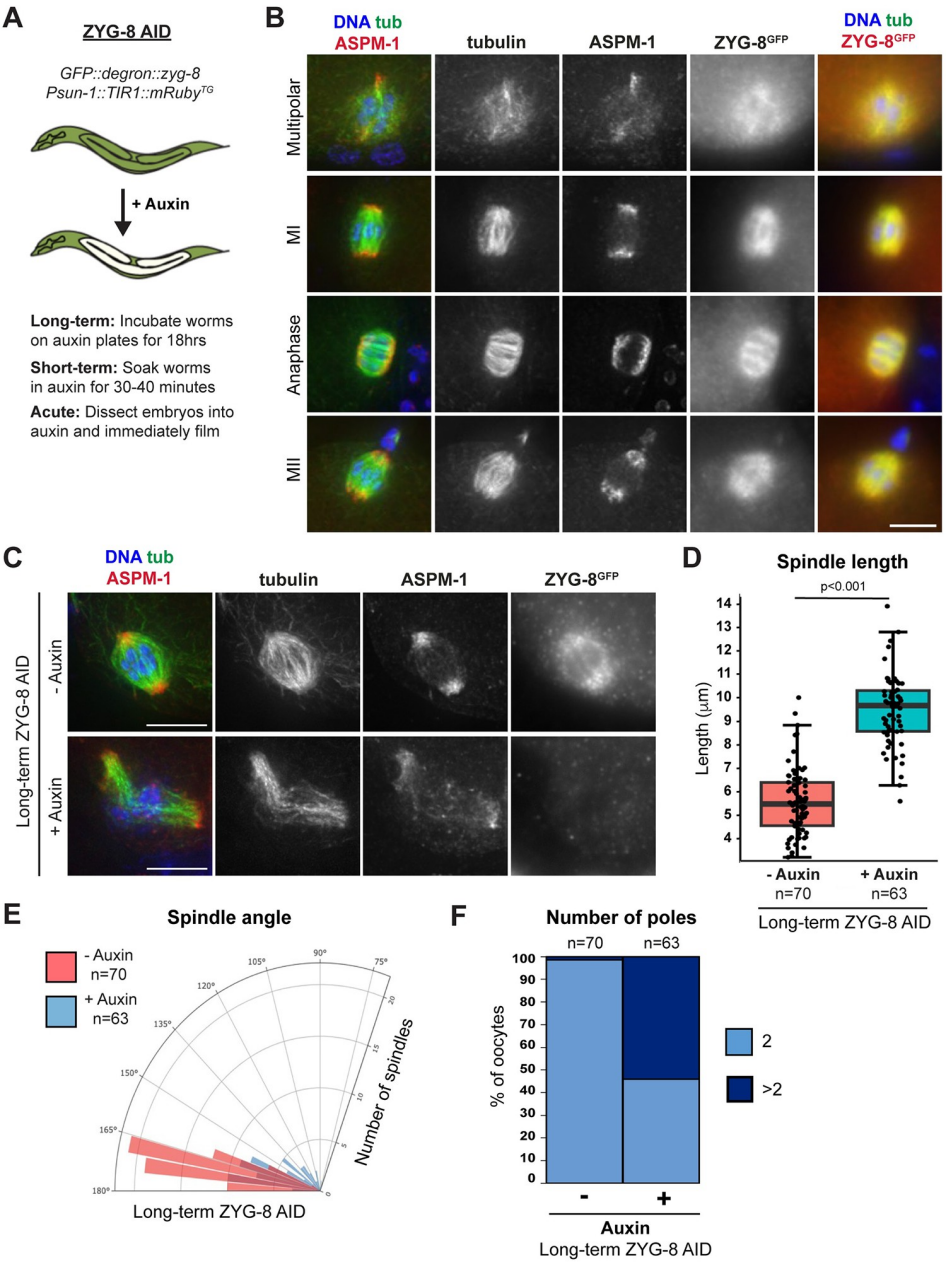
ZYG-8 localizes diffusely across the oocyte spindle and is required for proper spindle assembly. (A) Schematic representation of the auxin-inducible degradation system for ZYG-8 depletion in *C*. *elegans*. (B) Immunofluorescence images of oocytes in the ZYG-8 AID strain showing ZYG-8 localization throughout meiosis; shown are tubulin (green), DNA (blue), ASPM-1 (red, left panel), and ZYG-8 (stained using a GFP antibody; red, right panel). ZYG-8 is diffusely localized to the oocyte spindle throughout Meiosis I and II. Due to diffuse ZYG-8 localization, ZYG-8 images shown are not deconvolved. (C) Immunofluorescence images of either control spindles or spindles treated overnight on auxin-containing plates (“long-term AID”); shown are tubulin (green), DNA (blue), ASPM-1 (red), and ZYG-8 (stained using a GFP antibody). (D-F) Quantification of spindle length, spindle angle, and number of ASPM-1-marked poles. Following long-term AID, oocyte spindles were on average longer (p<0.001), more bent (p<0.001), and some had additional ASPM-1-marked poles. Scale bars = 5μm.

To investigate ZYG-8 further, we turned to the auxin-inducible degradation (AID) system, which enables spatially and temporally controlled depletion of proteins [5]. Using CRISPR/Cas9 technology, we introduced a GFP::degron tag onto the N-terminus of ZYG-8 in a strain expressing the ubiquitin ligase TIR1 from a germline-specific promoter (hereafter referred to as “ZYG-8 AID”; Fig 2A). To validate this strain, we incubated adult worms on auxin-containing plates for 18 hours (“long-term AID”) and verified that this treatment resulted in robust ZYG-8 depletion from the germ line using embryo-only western blotting (S2A Fig). Notably, under these conditions spindles in mitotically-dividing one-cell stage embryos were mispositioned, phenocopying defects observed in previous studies of *zyg-8* mutants (S2B Fig) [22,23]. Moreover, we analyzed the ZYG-8 AID strain in the absence of auxin, to determine if tagging ZYG-8 caused major phenotypes on its own. Notably, we did not detect any embryonic lethality in the ZYG-8 AID strain in the absence of auxin (<1% dead eggs) and the lengths and angles of oocyte spindles were similar to a control strain that expresses TIR1 but has untagged ZYG-8 (S2C and S2D Fig). Therefore, we set out to use this strain to study the role of ZYG-8 in oocytes.

Because ZYG-8 is tagged with GFP in the ZYG-8 AID strain, we first assessed ZYG-8 localization throughout all stages of meiosis in oocytes. ZYG-8 localization to microtubules begins during spindle assembly, with ZYG-8 localized diffusely across the entire meiotic spindle. ZYG-8 remains on the spindle through anaphase, and this pattern repeats in Meiosis II (Fig 2B). Notably, ZYG-8 staining was undetectable upon long-term AID, again confirming robust depletion (Fig 2C). Under these conditions, spindles were longer than they were in the absence of auxin (Fig 2D). Moreover, auxin-treated spindles were on average more bent than control spindles (Fig 2E) and some spindles contained more than two ASPM-1 clusters (Fig 2F), mirroring the phenotypes seen in the temperature-sensitive *zyg-8* mutants (Figs 1 **and** S1). As in our mutant analysis, we observed similar defects in Meiosis I and Meiosis II following ZYG-8 AID, so our quantifications include both stages. These results supported the hypothesis that ZYG-8 is required for normal spindle formation in oocytes and confirmed that the ZYG-8 AID strain could be used to further probe ZYG-8 function.

### ZYG-8 is required to maintain the stability of pre-formed oocyte spindles

**Fig 3:**
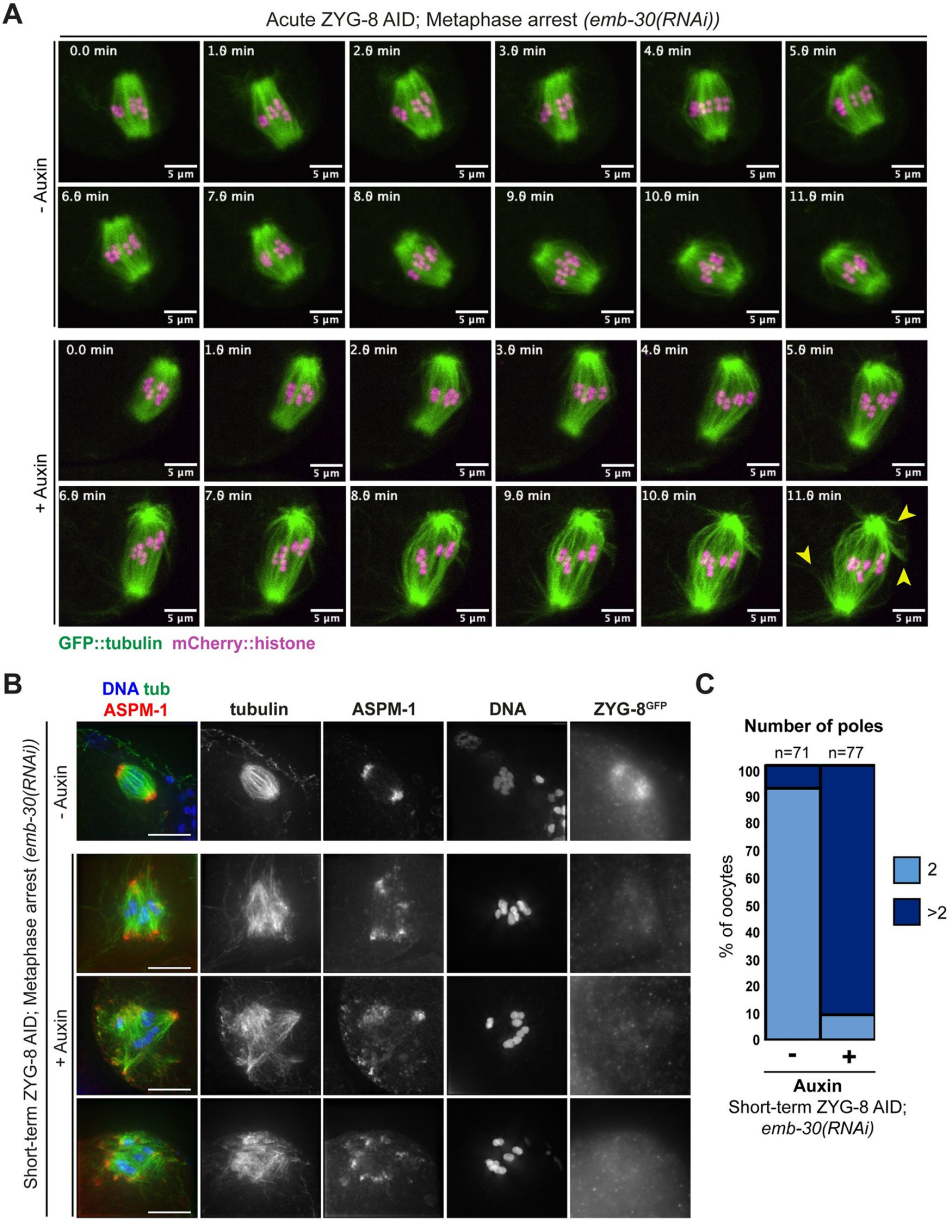
ZYG-8 depletion from pre-formed spindles results in spindle elongation and loss of bipolarity. (A) Live imaging of *emb-30(RNAi)* metaphase-arrested spindles; shown are GFP::tubulin (green) and mCherry::histone (magenta). In the absence of auxin (top panels), spindles maintain bipolarity and chromosome alignment throughout the time-lapse. In auxin treated oocytes (“acute AID”, bottom panels), the spindle elongates, microtubule bundles appear splayed away from the spindle center (arrowheads), and chromosomes become misaligned. (B) Immunofluorescence images of metaphase-arrested *(emb-30(RNAi))* spindles in the ZYG-8 AID strain. When worms were soaked in auxin for 30–40 minutes (“short-term AID”; rows 2–4), spindles became multipolar and chromosomes appeared more dispersed compared to control spindles (row 1). (C) Quantification of number of poles per spindle in control and short-term AID metaphase-arrested spindles. Scale bars = 5μm.

Since the AID system enables rapid protein depletion, we next sought to remove ZYG-8 from pre-formed spindles to test whether ZYG-8 is required to maintain proper spindle structure. To this end, we generated a version of the ZYG-8 AID strain expressing GFP::tubulin and mCherry::histone; using this strain we could dissect oocytes directly into auxin and visualize the effects of ZYG-8 depletion on the spindle in real time (“acute AID”). To enrich for pre-assembled bipolar spindles, we arrested oocytes in Metaphase I by depleting anaphase promoting complex (APC) component EMB-30. Without auxin, metaphase-arrested spindles in ZYG-8 AID oocytes maintain bipolarity (Fig 3A, S1 Video, 5/5 videos). In contrast, upon dissection into auxin, ZYG-8 AID oocyte spindles immediately began to elongate and microtubules appeared splayed away from the midspindle within a few minutes (Fig 3A, S2 Video; phenotypes seen in 10/10 videos).

To assess the consequences of more extended auxin treatment on pre-formed spindles, we soaked *emb-30(RNAi)* ZYG-8 AID worms in auxin for 30–40 minutes and then performed immunofluorescence (“short-term AID”); these conditions led to reduced ZYG-8 staining (Fig 3B). Although western blotting demonstrated that a small amount of ZYG-8 was still present following this depletion regime (S2A Fig), we nevertheless observed severe spindle defects (Fig 3B). Following auxin treatment, over 80% of spindles had 3 or more ASPM-1 clusters, reflecting pole fragmentation and/or loss of spindle bipolarity (Fig 3C); the severity of this phenotype precluded the measurement of spindle lengths, since it was difficult to define a primary spindle axis in most cases. Additionally, in contrast to control metaphase-arrested oocytes, we noticed that chromosomes were often misaligned after short-term ZYG-8 AID (Fig 3B). Combining the results of our live and fixed imaging, we propose that once ZYG-8 levels start to drop, the spindle begins to elongate and lose midspindle integrity and then the poles split and the spindle loses bipolarity.

To ensure that these spindle phenotypes were not artifacts caused by the *emb-30(RNAi)*-induced metaphase arrest, we performed both short-term and acute ZYG-8 depletion on unarrested oocytes. Following short-term AID depletion, we observed an increased number of spindles with more than two ASPM-1 clusters and a greater average spindle angle and length compared to untreated worms (S3A–S3D Fig). Moreover, we also observed defects when we performed acute ZYG-8 AID on unarrested oocytes (S3E Fig). While control spindles were able to maintain bipolarity and undergo anaphase (S3 Video, 5/5 videos), metaphase spindles exposed to auxin exhibited defects, including destabilization of the midspindle and spindle bending (S4 Video; phenotypes seen in 5/5 videos). Although we did not observe chromosome segregation during our imaging time course, a recent study observed bidirectional chromosome segregation upon ZYG-8 depletion, so we infer that these spindles would have initiated anaphase had we filmed longer [26].

Altogether, these data demonstrate that ZYG-8 is required for both the assembly and stability of acentrosomal spindles. However, we noted that the percentage of spindles with extra poles following short-term ZYG-8 AID in metaphase-arrested oocytes (70/77; 91%) was higher than in unarrested oocytes (10/44; 23%) (S3D Fig). Since the unarrested condition likely includes a proportion of spindles where ZYG-8 was depleted prior to or during spindle formation rather than in metaphase, this result could suggest that ZYG-8 plays a more important role in maintaining proper spindle structure than it plays during spindle assembly. This interpretation is also consistent with the observation that the phenotype observed in short term metaphase-arrested oocytes is more severe than following long-term AID (34/63, 54%), where ZYG-8 is depleted before spindle assembly in all oocytes (S3D Fig). However, it is also possible that the metaphase arrest alters spindle behavior in a manner that exacerbates the depletion phenotypes.

### ZYG-8 depletion triggers excess outward forces in the oocyte spindle

Next, we sought to understand why ZYG-8 depletion affected spindle pole organization. One possibility is that the observed pole splitting could be a consequence of global effects on the spindle; perhaps splaying of microtubules in the midspindle disrupts the overlap zone and subsequently causes the spindle to lose bipolarity. Alternatively, ZYG-8 may play a direct role in forming and/or stabilizing the poles themselves. To explore these possibilities, we sought to deplete ZYG-8 and assess the ability of monopolar spindles to form; these spindles have an organized pole but lack a central overlap region.

**Fig 4:**
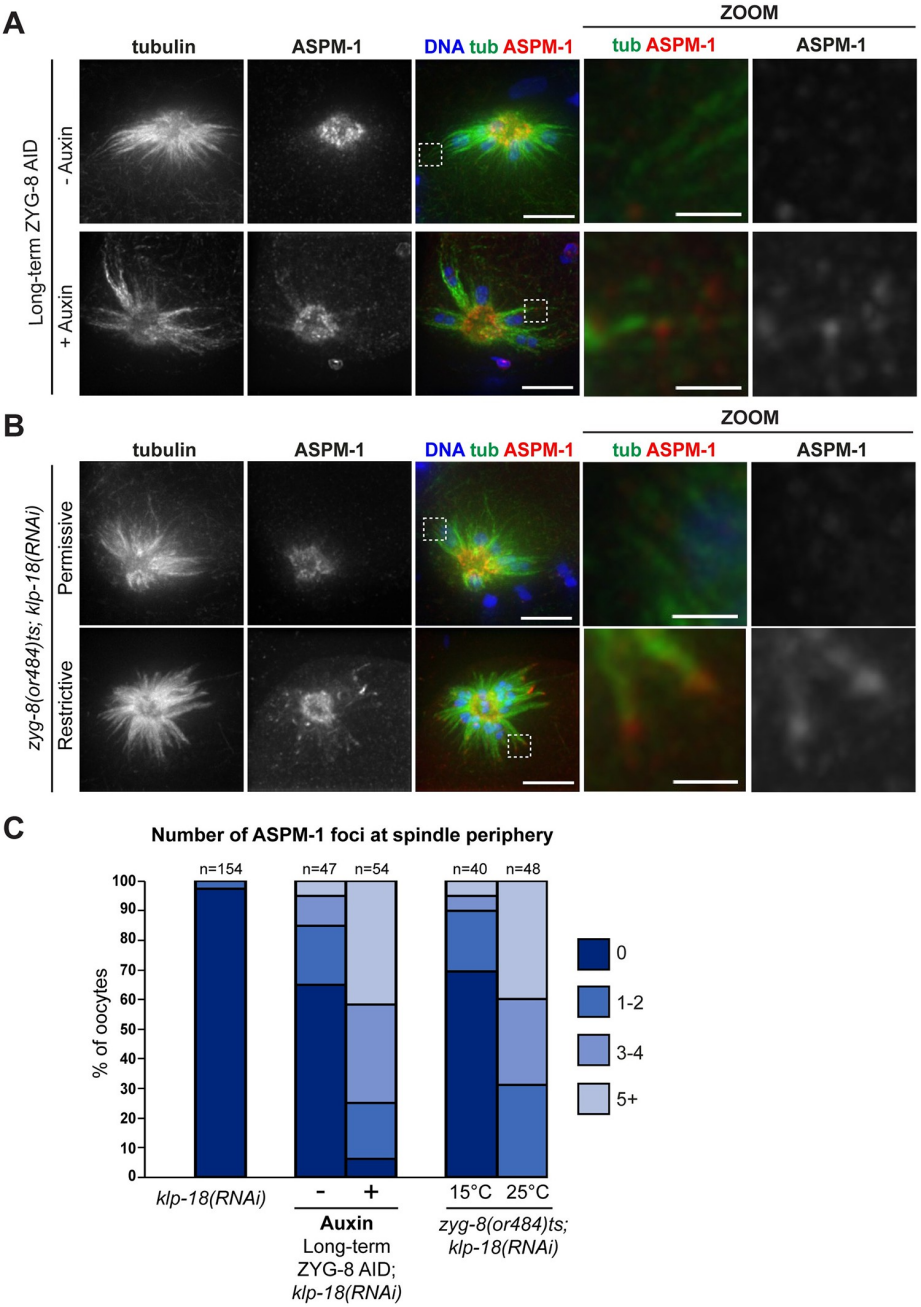
ZYG-8 depletion causes minus ends to be sorted outwards in the absence of KLP-18. (A) Immunofluorescence images of *klp-18(RNAi)* oocytes in the ZYG-8 AID strain. Shown are tubulin (green), DNA (blue), and ASPM-1 (red). In the absence of auxin, a monopolar spindle forms with ASPM-1-marked microtubule minus ends concentrated in the center (top row). Upon long-term AID to deplete ZYG-8, a central ASPM-1-marked pole still forms but there is also ASPM-1 localization at the end of microtubule bundles at the spindle periphery (zoom), suggesting that minus ends are being sorted outwards despite the loss of KLP-18. (B) Immunofluorescence images of *klp18(RNAi)* oocytes at either the permissive (15°C) or restrictive (25°C) temperature in the *zyg-8(or484)* temperature sensitive mutant. Shown are tubulin (green), DNA (blue), and ASPM-1 (red). After incubation at the restrictive temperature, monopolar spindles have ASPM-1 localized to the periphery of the spindle at the end of microtubule bundles (zooms). Scale bar = 5μm (regular images); 1μm (zooms). (C) Quantification of number of ASPM-1 foci seen at the periphery of each monopolar spindle.

Following depletion of KLP-18/kinesin-12 via RNAi, microtubule minus ends are not sorted outwards during spindle assembly, so a monopolar spindle forms with microtubule minus ends in the center and microtubule plus ends oriented towards the periphery of the aster [10,13] (Fig 4A and 4C). When we combined *klp-18(RNAi)* with long-term ZYG-8 AID, monopolar spindles were still able to form with microtubule minus end marker ASPM-1 concentrated at the single pole, suggesting that ZYG-8 is not required to form an organized pole structure. Thus, the pole defects observed in our previous experiments, when KLP-18 was not depleted, are likely due to global effects on the spindle. However, the monopolar spindle experiment also revealed a surprising phenotype; ASPM-1 was often present at the periphery of the aster on the ends of microtubule bundles, suggesting that some microtubule minus ends were sorted outwards during monopolar spindle assembly (Fig 4A and 4C). We observed the same phenotype when we performed *klp-18(RNAi)* on the *zyg-8(or484)* temperature-sensitive mutant and then incubated it at the restrictive temperature; monopolar spindles formed that contained ASPM-1-marked minus ends at the periphery (Fig 4B and 4C). Thus, ZYG-8 inactivation enables some microtubule minus ends to be sorted outwards during spindle assembly in oocytes lacking KLP-18, suggesting that, unlike the *klp-18(RNAi)* alone condition, outward forces were being generated. Thus, ZYG-8 may regulate forces in the oocyte spindle.

**Fig 5:**
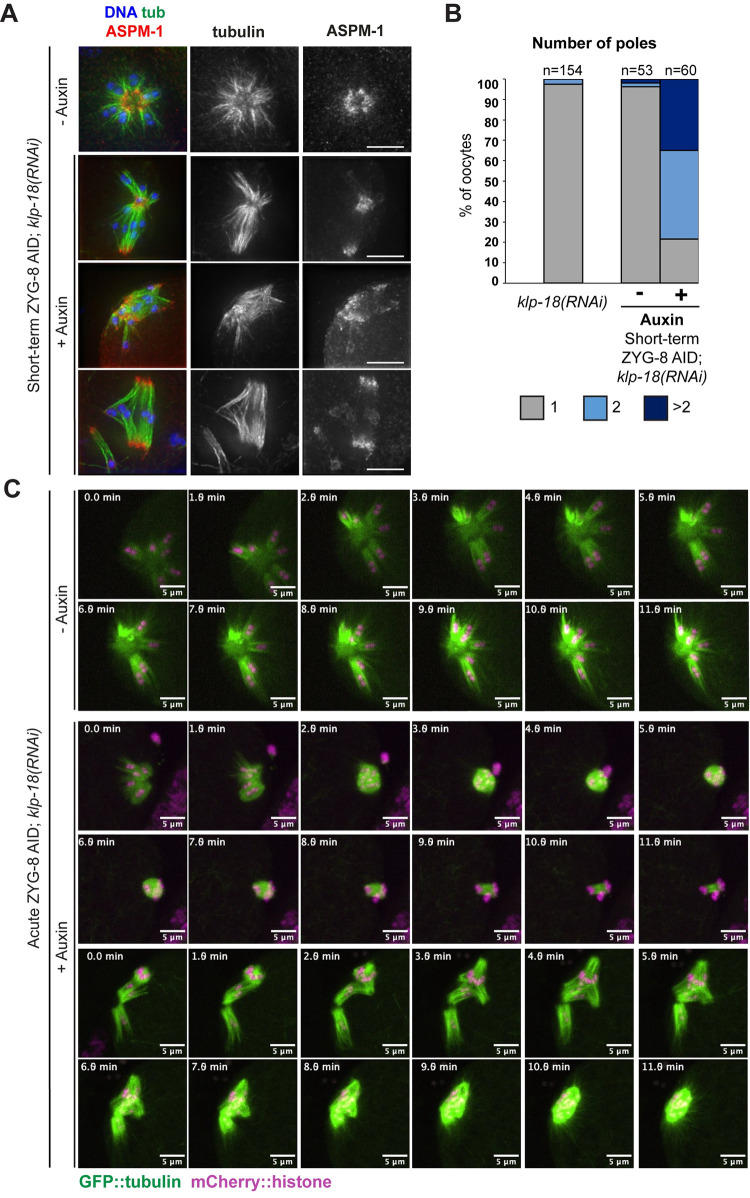
Removal of ZYG-8 from pre-formed monopolar spindles triggers spindle reorganization and can restore bipolarity. (A) Immunofluorescence images of ZYG-8 AID, *klp-18(RNAi)* oocytes. Shown are tubulin (green), DNA (blue), and ASPM-1 (red). Upon short-term auxin treatment to deplete ZYG-8 from pre-formed monopolar spindles, microtubule minus ends were no longer organized into a single pole, suggesting that microtubule minus ends were sorted outwards; in some cases, bipolarity appeared to be largely restored (bottom row). (B) Quantification of the number of spindle poles from the experiment shown in A. (C) Live imaging of *klp-18(RNAi)*, ZYG-8 AID spindles; shown are GFP::tubulin (green) and mCherry::histone (magenta). Control monopolar spindles remain monopolar as chromosomes slowly move towards the center pole in anaphase (rows 1–2). When treated with auxin, monopolar spindles reorganize (rows 3–6). In some cases, these spindles were able to segregate chromosomes bidirectionally, suggesting that bipolarity was restored (rows 3–4). We also observed cases where a monopolar MII spindle reorganized and appeared to incorporate the polar body, ultimately forming what appeared to be a bipolar spindle (rows 5–6). Scale bars = 5μm.

To investigate this possibility, we sought to remove ZYG-8 from pre-formed *klp-18(RNAi)* monopolar spindles to determine whether this would result in the activation of outward forces. Remarkably, when we performed short-term ZYG-8 AID on *klp-18(RNAi)* monopolar spindles, we saw a loss of monopole integrity in most oocytes (47/60; 78%) and spindles were sometimes able to restore bipolarity (26/60, 43%; Fig 5A and 5B). We also observed the same phenotype using live imaging. It has been previously shown that during monopolar anaphase, chromosomes move towards the center of the aster as the spindle shrinks [27]; we observed this same behavior in the ZYG-8 AID strain in the absence of auxin (Fig 5C, S5 Video). In contrast, following acute ZYG-8 AID monopolar spindles began to reorganize (12/12 videos), and these spindles were sometimes able to reestablish bipolarity and segregate chromosomes bidirectionally (4/12 videos; Fig 5C, S6 and S7 Videos). Thus, ZYG-8 depletion appears to activate outward forces in the oocyte spindle. Mirroring our findings with bipolar spindles, it is notable that ZYG-8 depletion from pre-formed monopolar spindles (short-term and acute AID; Fig 5) had a more severe phenotype than when ZYG-8 was depleted prior to monopole assembly (long-term AID; Fig 4). This finding again suggests that ZYG-8 may play a more important role in maintaining proper spindle morphology during metaphase than it plays during spindle assembly.

### With loss of BMK-1 and KLP-18, outward forces are no longer activated upon ZYG-8 depletion

**Fig 6:**
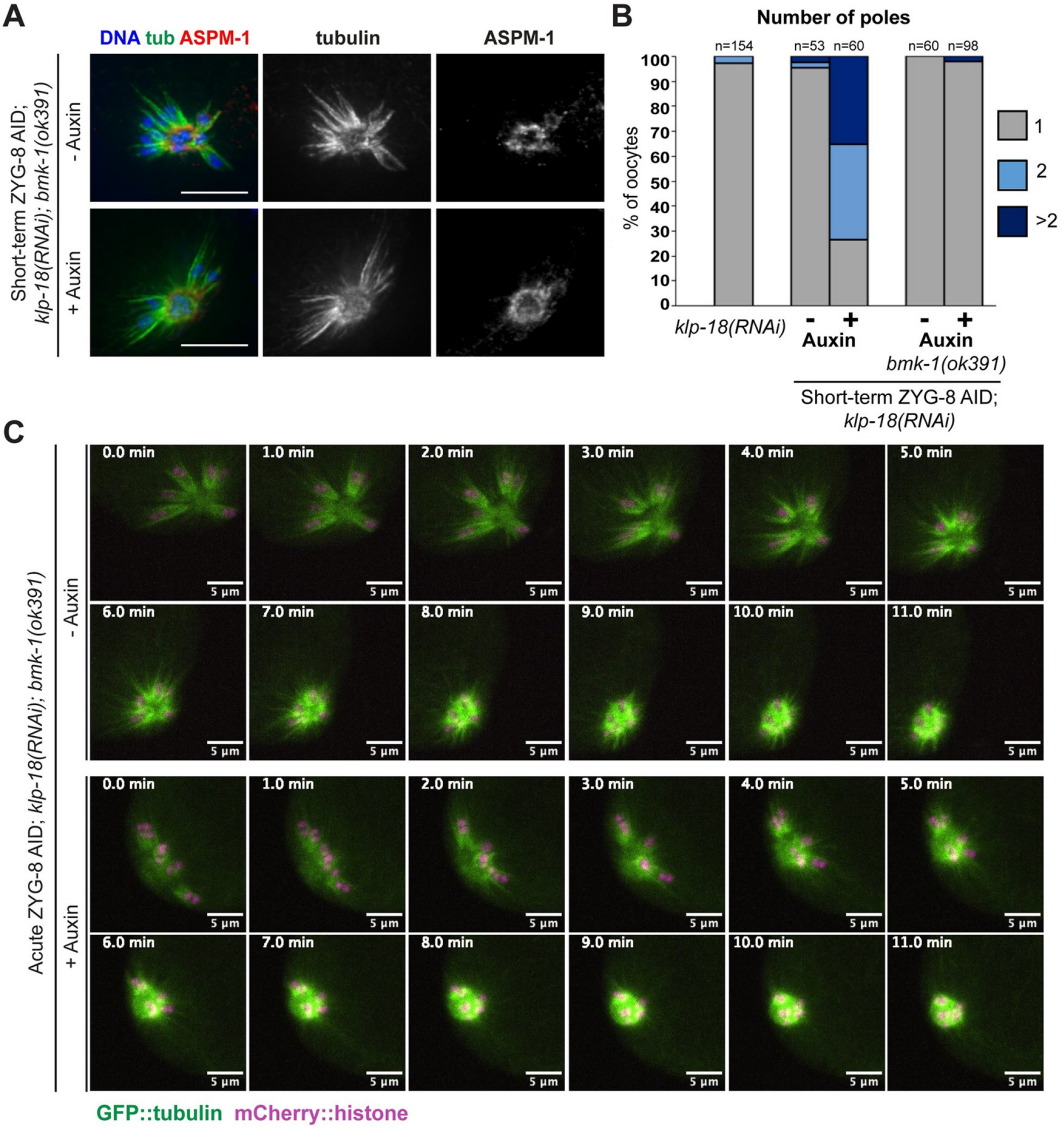
With loss of BMK-1, monopolar spindles do not reorganize upon ZYG-8 removal. (A) Immunofluorescence images of *klp-18(RNAi)* in ZYG-8 AID, *bmk-1(ok391)* oocytes. Shown are tubulin (green), DNA (blue), and ASPM-1 (red). In this strain, monopolar spindles did not reorganize upon short-term auxin treatment. (B) Quantification of the experiment shown in A. Note that the data from the ZYG-8 AID strain without the *bmk-1* mutation is repeated from Fig 5B, for purposes of comparison. (C) Live imaging of *klp-18(RNAi)* in the ZYG-8 AID, *bmk-1(ok391)* strain; shown are GFP::tubulin (green) and mCherry::histone (magenta). In both the presence and absence of auxin, spindles remain monopolar as chromosomes slowly move towards the center pole in anaphase and the monopolar spindle shrinks, similar to what has been reported for *klp-18(RNAi)* alone [27]. Scale bars = 5μm.

The discovery that outward forces were generated following double depletion of KLP-18 and ZYG-8 raised the question of what factor could be providing those forces. Although KLP-18 is the major outward force generating motor in *C*. *elegans* oocytes, we recently reported that BMK-1/kinesin-5 provides an outward sorting force that is not detected when KLP-18 is present [8]. Therefore, we reasoned that BMK-1 could be producing the outward forces generated when ZYG-8 is depleted. To investigate this hypothesis, we generated a version of the ZYG-8 AID strain that also contained a loss of function *bmk-1(ok391)* mutation [15]. We then performed *klp-18(RNAi)* combined with short-term ZYG-8 AID, to determine if outward forces were still activated if BMK-1 function was compromised. Strikingly, with the loss BMK-1, monopoles remained intact following ZYG-8 depletion (Fig 6A and 6B), suggesting that outward forces were not generated. This phenotype was also confirmed using live imaging, where auxin-treated ZYG-8 AID monopolar spindles did not reorganize into bipolar spindles in the *bmk-1(ok391)* mutant and we never observed chromosomes segregating bidirectionally in anaphase; instead chromosomes moved towards the center of the monopole as the spindle decreased in size, indistinguishable from normal monopolar anaphase (Fig 6C, S8 and S9 Videos; 5/5 auxin-treated spindles remained monopolar). These data demonstrate that BMK-1 produces the outward forces that are activated upon ZYG-8 and KLP-18 co-depletion and raise the possibility that ZYG-8 regulates BMK-1 either directly or indirectly. This hypothesis is consistent with the phenotypes observed following ZYG-8 depletion and in the *zyg-8ts* mutants; if ZYG-8 normally dampens BMK-1 activity, then ZYG-8 inhibition would generate BMK-1-driven outward forces that could cause the spindle to elongate and disrupt its organization.

However, while our findings implicate ZYG-8 in regulating BMK-1, they do not rule out the possibility that ZYG-8 has other functions as well. To test this, we assessed the effects of depleting ZYG-8 in the *bmk-1(ok391)* strain without depleting KLP-18. Interestingly, we found that spindles were longer in the ZYG-8 AID; *bmk-1(ok391)* strain in the presence of auxin (S4A and S4B Fig). Moreover, some spindles had fragmented and/or multiple poles (S4C Fig). Therefore, the phenotypes observed following ZYG-8 depletion cannot be explained solely by overactivation of BMK-1, demonstrating that ZYG-8 contributes to proper spindle organization in additional ways.

### ZYG-8 is required for proper microtubule dynamics in acentrosomal spindles

Previous studies have demonstrated that ZYG-8 regulates microtubule assembly in *C*. *elegans* mitotically-dividing embryos; ZYG-8 depletion via RNAi led to reduced microtubule growth rates and increased nucleation rates [22,24]. We therefore reasoned that another function of ZYG-8 in oocytes could be to regulate microtubule dynamics within the spindle. To investigate this possibility, we performed fluorescence recovery after photobleaching (FRAP) to assess the turnover of microtubules in acentrosomal spindles formed with and without ZYG-8.

**Fig 7:**
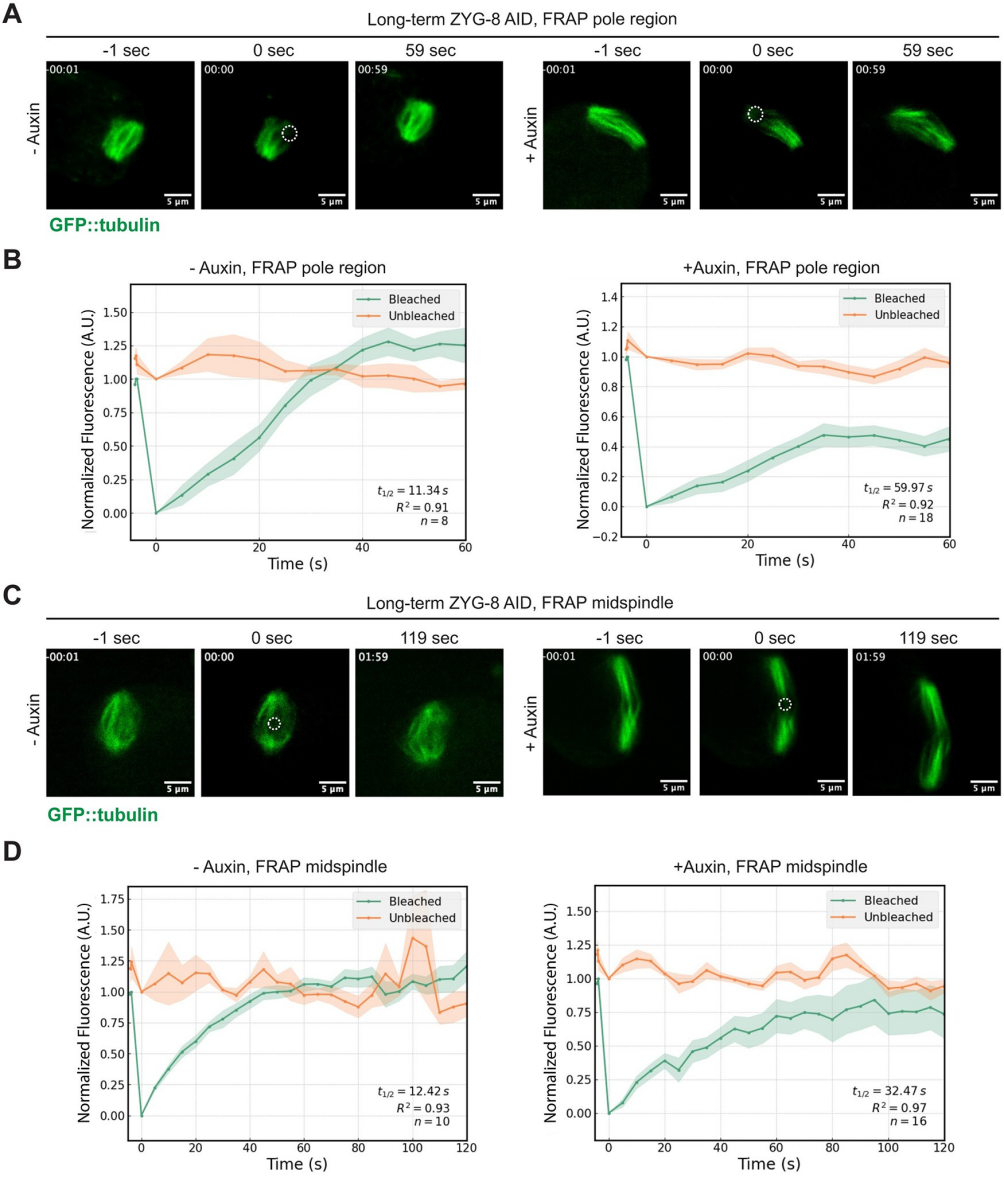
Loss of ZYG-8 decreases the rate of tubulin turnover at the spindle poles and spindle center. Fluorescence recovery after photobleaching (FRAP) was performed in ZYG-8 AID oocytes expressing GFP::tubulin. (A&C) Stills from FRAP movies; 3 frames were acquired before bleaching and then bleaching occurred at t = 0. The laser was focused either near the spindle pole (A) or near the spindle center (C). The white dotted circles denote the ROIs within the bleached region where fluorescence intensity was measured. (B&D) Graphs showing GFP::tubulin intensity throughout the FRAP timecourse. Bleached ROIs are represented with the green traces, and reference (unbleached) regions are represented with the orange traces; for (B) the reference ROI was obtained at the unbleached pole, and for (D) the reference ROI was an unbleached region near the center of the spindle. The solid lines are the average, and the standard error of the mean is shaded. t½ was calculated from fitting the recovery curve to a single exponential function. Spindles lacking ZYG-8 (following long-term AID) have a slower rate of GFP::tubulin fluorescence recovery in both the pole and midspindle regions. Time elapsed shown in (min):(sec). Scale bar = 5μm.

A previous study used FRAP to assess the turnover of GFP::tubulin at acentrosomal spindle poles [9]; we measured a similar rate of fluorescence recovery at poles in our ZYG AID strain without auxin (t ½ = 11.34 s) (Fig 7A and 7B **and** S10 Video). Strikingly, when we performed FRAP on spindle poles following long-term auxin treatment, we measured a much slower recovery time (t ½ = 59.97 s) (Fig 7A and 7B **and** S11 Video). Similarly, there were also effects on tubulin turnover in the center of the spindle following loss of ZYG-8. Without auxin, the GFP::tubulin signal was able to fully recover at rates similar to the poles (t ½ = 12.42 s), while recovery was slower in spindles following long-term AID (t ½ = 32.47 s) (Fig 7C and 7D, S12 and S13 Videos). To ensure that auxin itself was not causing these changes in microtubule dynamics, we exposed a strain that expressed TIR1 but lacked tagged ZYG-8 to long-term auxin treatment and then performed FRAP. Fluorescence recovery in this auxin-treated strain was similar to untreated worms (t ½ = 13.39 s) (S5 Fig), confirming that the effects on tubulin dynamics in the ZYG-8 AID strain were caused by loss of ZYG-8. The decreased turnover of GFP::tubulin suggests that microtubules are more stable following ZYG-8 depletion. This change in dynamics could contribute to the observed spindle defects since a global stabilization of microtubules would be predicted to cause spindle elongation. Thus, ZYG-8 is required for proper microtubule dynamics in the acentrosomal oocyte spindle, revealing another function of ZYG-8 that likely contributes to spindle assembly and stability.

### ZYG-8’s kinase activity is required for its function in meiosis and mitosis

**Fig 8:**
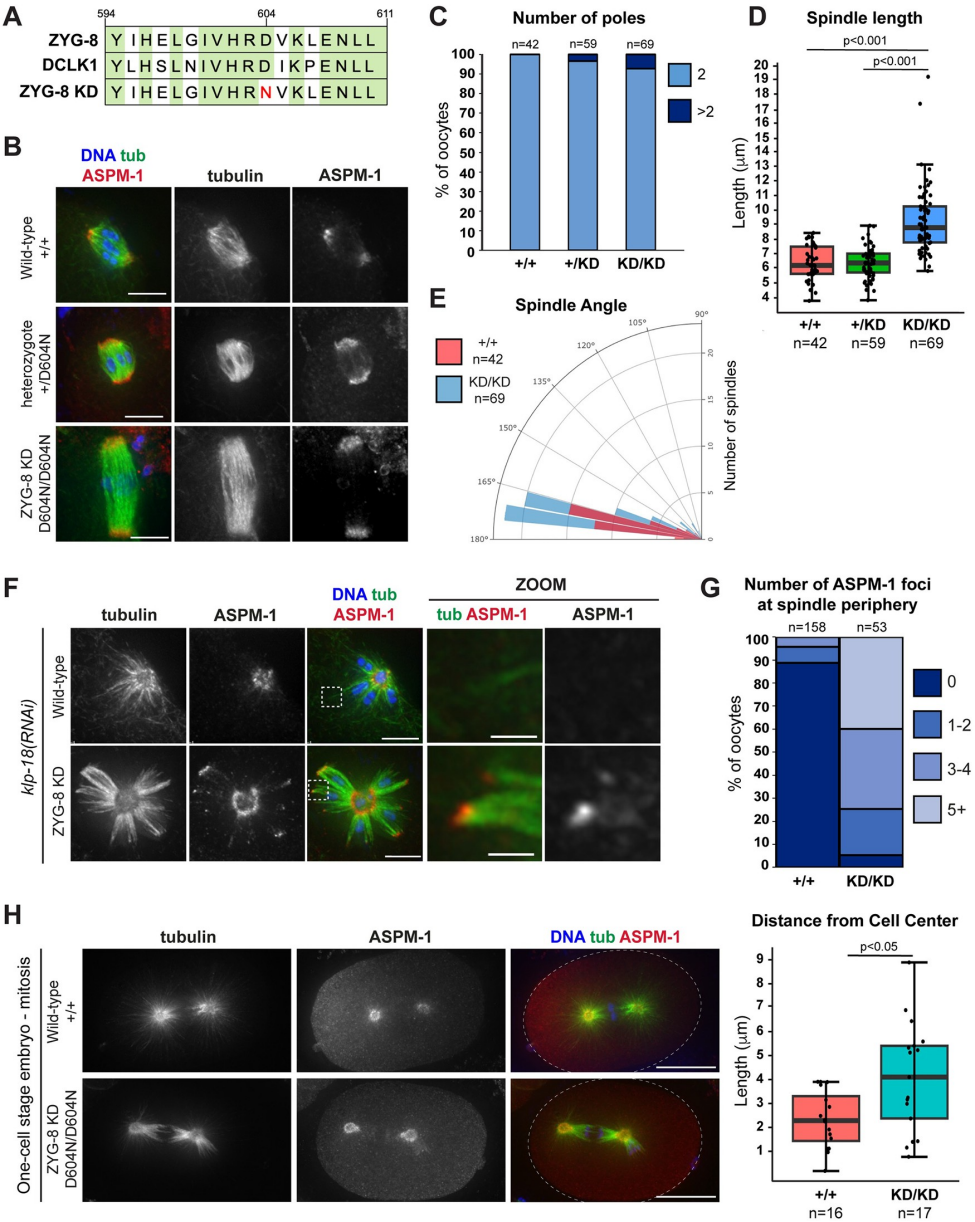
Kinase activity is required for ZYG-8 function in meiosis and mitosis. (A) Sequence of a portion of the ZYG-8 kinase domain, indicating the D604N “kinase dead” mutation (ZYG-8 KD). Green represents homology between ZYG-8 and its mammalian homolog DCLK1. (B) Immunofluorescence images of spindles in wild-type oocytes (row 1) and oocytes from *zyg-8*^*D604N*^ heterozygous (row 2) and homozygous (ZYG-8 KD, row 3) parents; shown are tubulin (green), DNA (blue), and ASPM-1 (red). Oocyte spindles from wild-type and heterozygous *zyg-8*^*D604N*^ parents were indistinguishable while spindles from homozygous *zyg-8*^*D604N*^ parents were longer in length. (C-E) Quantification of the number of ASPM-1-marked poles, spindle length, and spindle angle for the experiment shown in B. Spindles from homozygous *zyg-8*^*D604N*^ parents were longer than wild-type spindles (p<0.001) but there was no significant difference in the spindle angle (p>0.1). (F) Immunofluorescence images *of klp-18(RNAi)* monopolar spindles in oocytes from homozygous *zyg-8*^*D604N*^ parents (ZYG-8 KD, row 2) compared to wild type (row 1); shown are tubulin (green), DNA (blue), and ASPM-1 (red). ASPM-1 was present at the end of microtubules bundles at the periphery of monopolar spindles in the ZYG-8 KD strain (zooms). (G) Quantification of the number of ASPM-1 foci at the monopolar spindle periphery. (H) Immunofluorescence images of one-cell stage mitotic spindles in embryos from wild-type worms (row 1) and from homozygous *zyg-8*^*D604N*^ parents (row 2). Spindles in the ZYG-8 KD mutant are mis-positioned, phenocopying defects seen in *zyg-8* mutants [22,23,67] and following ZYG-8 AID. Quantification reflects the distance measured from the spindle center to the cell center (p<0.05). Scale bars in B and F = 5μm (regular images); 1μm (zooms). Scale bar in H = 10μm.

Finally, we sought to understand more about the mechanisms by which ZYG-8 functions. The mammalian homolog of ZYG-8, DCLK1 (doublecortin-like kinase 1), is upregulated in multiple cancers and there are ongoing efforts to generate inhibitors targeting its kinase activity as a therapeutic strategy [28–32]. However, whether the kinase activity of DCLK1 or any of its paralogs is required during cell division is not known. Therefore, to determine if ZYG-8’s kinase activity is required for its function, we used CRISPR to mutate a conserved aspartic acid within the catalytic loop of ZYG-8 to asparagine (D604N). This mutation was previously shown to render DCLK1 kinase dead [33,34] and we presumed that it would hamper ZYG-8 as well given high conservation in the kinase domain between the two proteins (Fig 8A). Homozygous mutant worms carrying this “kinase-dead” (“KD”) mutation laid 98% dead eggs, demonstrating that this mutation causes embryonic lethality.

To assess the effect of this mutation on meiotic spindle formation, we compared spindle morphology in oocytes from *zyg-8*^*D604N*^ heterozygous and homozygous parents to wild-type worms. While oocyte spindles in heterozygotes were approximately the same length as wild-type spindles, homozygous *zyg-8*^*D604N*^ oocyte spindles were substantially longer (Fig 8B and 8D). Similarly, when we performed *klp-18(RNAi)*, monopolar spindles were able to form in homozygous *zyg-8*^*D604N*^ oocytes, but over 90% of the spindles had ASPM-1 staining at the periphery of the aster (Fig 8F and 8G). These phenotypes were reminiscent of the spindle assembly phenotypes observed in the *zyg-8* temperature-sensitive mutant strains and following long-term ZYG-8 AID (Figs 1, 2 and 4 **and** S1), suggesting that ZYG-8’s kinase activity is required for ZYG-8 function. Notably, we also observed mitotic defects in one-cell stage embryos from *zyg-8*^*D604N*^ homozygous parents (Fig 8H). Bipolar spindles formed but they were mispositioned, phenocopying defects seen in previous analysis of *zyg-8* temperature sensitive mutants [22,23] and following ZYG-8 AID (S2B Fig). Thus, kinase activity is required for ZYG-8 function in mitosis as well.

Interestingly, however, we noticed that spindles in homozygous *zyg-8*^*D604N*^ oocytes were not as severely disrupted as those in the *zyg-8ts* or ZYG-8 AID strains. We did not observe substantial fragmentation of spindle poles in the *zyg-8*^*D604N*^ mutant (Fig 8C) and the spindle angles were similar to wild type (Fig 8E). This suggests that while kinase activity is required to prevent spindle over-elongation, other regions of the protein, such as the doublecortin domain, may function to stabilize the bipolar spindle structure to prevent pole and midspindle destabilization. In a similar vein, since our data suggest that ZYG-8 regulates both microtubule dynamics and motor-driven spindle forces, it is also possible that this represents a separation of function mutation, and that kinase activity is required for one of these functions but not the other. Taken together, our findings demonstrate that ZYG-8 plays multiple important roles during meiosis and provide important new insights into how the spindle is formed and stabilized in oocytes.

## Discussion

### ZYG-8 plays multiple roles in forming and stabilizing the acentrosomal spindle

**Fig 9:**
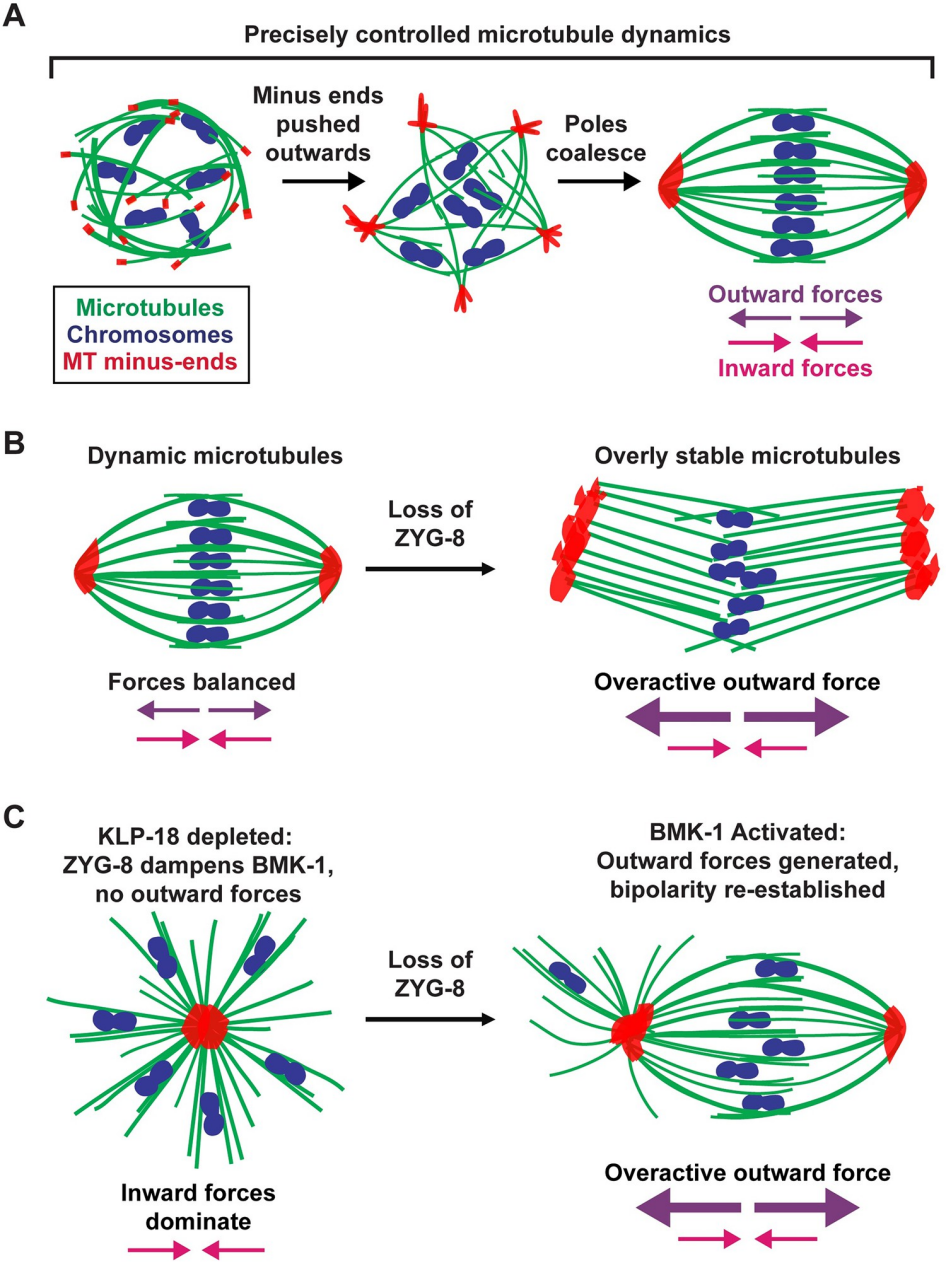
Model: ZYG-8 is required for proper force balance and microtubule dynamics in the acentrosomal spindle. Chromosomes (blue), microtubules (green), and microtubule minus ends (red). (A) As an acentrosomal spindle forms, microtubules begin to nucleate in the presence of condensed chromosomes. KLP-18 (kinesin-12) provides the major outward force and sorts microtubule minus ends to the outer periphery of the forming spindle. Minus ends then form multiple poles that coalesce into two distinct poles at either end of the spindle. In a bipolar spindle, the outward and inward forces are balanced. Moreover, microtubule dynamics must be precisely controlled throughout the meiotic divisions. (B) Upon removal of ZYG-8 from a balanced metaphase spindle, microtubules become more stable and outward forces become overactive, pushing minus ends outward and elongating the spindle. (C) When ZYG-8 is removed from a monopolar spindle lacking KLP-18, BMK-1 becomes over-activated and microtubule minus ends are sorted to the periphery of the spindle, reestablishing bipolarity.

Altogether, our data reveal an important role for ZYG-8 in facilitating the assembly and stability of the oocyte spindle (Fig 9). During spindle assembly, microtubule minus ends must be pushed outwards away from the chromosomes, necessitating the production of outward forces [10]. However, mechanisms must also exist to limit these forces and microtubule dynamics must be precisely regulated in order for the spindle to maintain a stable length (Fig 9A). Our studies support a model in which ZYG-8 regulates both microtubule dynamics and motor-driven forces, to promote proper spindle size and organization.

A role in regulating microtubule dynamics is supported by our FRAP experiments; ZYG-8 depletion slowed the turnover of tubulin throughout the spindle, suggesting that microtubules became more stable. This could be a reason why spindles formed in the absence of ZYG-8 are longer than in wild type oocytes, and why acute removal of ZYG-8 from pre-formed spindles triggers spindle elongation; globally stabilizing microtubules could cause the spindle to increase in size (Fig 9B). However, we also found evidence that ZYG-8 plays an additional role in regulating spindle forces, which could also impact spindle length. Specifically, experiments with monopolar spindles revealed that ZYG-8 depletion results in excess outward forces during spindle assembly and ZYG-8 removal from pre-formed monopoles resulted in minus ends being pushed outwards to form multiple poles (Fig 9C). Remarkably, in the latter experiment, monopolar spindles were sometimes able to convert back to bipolar spindles that could mediate bi-directional chromosome segregation. These findings suggest that motor-driven outward forces are also activated following ZYG-8 depletion (Fig 9B). Consistent with this interpretation, when we removed ZYG-8 from monopolar spindles in *bmk-1* mutants, the monopoles did not reorganize. This result demonstrated that BMK-1 provided the outward force that drove spindle bipolarization upon ZYG-8 depletion, and suggested that ZYG-8 may normally dampen the activity of BMK-1. Thus, we propose that ZYG-8 plays at least two important roles in *C*. *elegans* oocytes: 1) ZYG-8 regulates microtubule dynamics, to facilitate the proper turnover of microtubules within the spindle, and 2) ZYG-8 influences the activity of motors that walk along those microtubules, to tune the forces that build and stabilize the spindle. These two functions could be interconnected. For example, perhaps stabilizing microtubules enables motors to exert excess forces; therefore, by promoting microtubule turnover within the spindle, ZYG-8 would also indirectly dampen the activity of motors. However, it is also possible that these are separable functions and that ZYG-8 could regulate motors directly. Future experiments will be important to explore these possibilities. Notably, both of these proposed functions are consistent with ZYG-8’s localization pattern; ZYG-8 localizes broadly across the spindle, which would enable it to modulate microtubule dynamics at both the poles and midspindle, and it also co-localizes with BMK-1 throughout meiosis, making it plausible that it could also regulate the activity of this motor.

In addition to spindle elongation, we also observed pole defects upon ZYG-8 depletion; spindles formed in the absence of ZYG-8 sometimes had fragmented or multiple poles. These defects are similar to those previously observed upon dynein depletion, which caused spindle poles to become broad and unfocused [8,35]. This raises the possibility that ZYG-8, like dynein, may play a direct role in stabilizing acentrosomal poles. However, we do not favor this interpretation for a few reasons. First, while dynein removal does not disrupt the central microtubule overlap region of the spindle [8], ZYG-8 depletion causes midspindle bending, suggesting that ZYG-8 does not solely function at poles. Second, monopolar spindles were able to form following double depletion of KLP-18 and ZYG-8; these spindles had an organized central pole, suggesting that ZYG-8 is not required for pole formation *per se*. Finally, when ZYG-8 was removed from pre-formed monopolar spindles, the microtubule minus ends that were pushed outwards were able to form poles. This phenotype stands in stark contrast to that seen following dynein depletion; when dynein was removed from pre-formed monopolar spindles, the entire monopole came apart, dispersing meiotic chromosomes throughout the cytoplasm [8]. Thus, we infer that ZYG-8 is unlikely to play the same role as dynein in stabilizing poles directly, and instead we suggest that it plays a more global role in forming and stabilizing acentrosomal spindles. One possibility is that the pole defects could be caused by a combination of the proposed effects on microtubule dynamics and spindle forces; if microtubules become more stable and motors produce excess outward forces, this may cause stress on the spindle structure that could cause the midspindle to bend and the poles to split.

Interestingly, the phenotypes we observed were more severe when we removed ZYG-8 from pre-formed spindles than when we depleted ZYG-8 prior to spindle assembly. Spindles formed in the absence of ZYG-8 often had split poles or were multipolar, consistent with a role for ZYG-8 in spindle assembly. However, the percentage of multipolar spindles was even higher when ZYG-8 was removed from metaphase-arrested spindles; we speculate that the arrest may be exacerbating this phenotype by pausing spindles in metaphase where they may be subjected to prolonged spindle forces. Similarly, monopolar spindles formed in the absence of ZYG-8 had some minus ends at the periphery of the spindle, but an organized central pole was able to form. However, removal of ZYG-8 after monopole formation caused spindle reorganization, both with and without a metaphase arrest. Together, these findings suggest that ZYG-8 may play a more significant role in stabilizing the metaphase spindle than it does in forming the spindle.

### Potential functions of ZYG-8’s kinase activity

An important question is how ZYG-8 performs its various roles within the spindle. Notably, our studies demonstrate that kinase activity is required for ZYG-8 function. This is an important finding, since the mammalian homolog of ZYG-8 (DCLK1/doublecortin-like kinase 1) is upregulated in a wide range of cancers, and knockdown studies have suggested that it is required for tumor progression. Because of this, there has been a recent effort to find small molecules that inhibit DCLK1’s kinase activity, to be used as therapeutics [36–42]. However, whether kinase activity is essential for DCLK1’s function in cell division was not previously known. Here, we show that kinase activity is important for the function of a DCLK1-family protein during both mitosis and meiosis in an *in vivo* model, providing evidence that may validate this therapeutic strategy.

How kinase activity regulates ZYG-8 function is an important area of future study. Given that ZYG-8 depletion appears to activate outward forces, one possibility is that ZYG-8 phosphorylates motors directly. There is a large amount of evidence, spanning multiple species and cell types, that kinesin motors can be regulated by phosphorylation [43–48]. For example, phosphorylation of Kinesin-5 motors can impact localization, motor activity, or function in mitosis [49]. In *C*. *elegans*, Aurora B kinase (AIR-2) is required for BMK-1’s localization to mitotic and meiotic spindle microtubules *in vivo* and can phosphorylate BMK-1’s tail domain *in vitro*, suggesting that phosphorylation may regulate its localization [15]. In *Xenopus laevis* egg extract, Cdk1 phosphorylation increases kinesin-5 binding to microtubules [50] and in *D*. *melanogaster*, kinesin-5 was shown to undergo a reversible phosphorylation in order to activate or deactivate the motor [51]. It has also been shown that several kinesin families can be regulated by autoinhibition controlled by phosphorylation; this may be a general mechanism for kinesin regulation [46]. Given these established roles for phosphorylation in modulating motor activity, it is possible that ZYG-8 phosphorylates BMK-1 and/or other motors to regulate force balance.

Similarly, it is possible that ZYG-8 could phosphorylate factors that regulate microtubule dynamics. Notably, DCLK1 has been shown to phosphorylate a microtubule associated protein (MAP7D1) to facilitate axon elongation in mouse cortical neurons [52]; it is possible that ZYG-8 could phosphorylate microtubule regulatory factors in *C*. *elegans* oocytes in a similar manner to modulate microtubule dynamics within the spindle. The microtubule depolymerase MCAK is known to be regulated by phosphorylation in multiple organisms [53] including *C*. *elegans* [17]. Moreover, members of the XMAP215 family of microtubule polymerases can also be phosphorylated [54] and the *C*. *elegans* XMAP215 homolog ZYG-9, like ZYG-8, is required for spindle stability in oocytes [9].

Or finally, another possibility is that ZYG-8’s kinase activity is required for its own localization or function. DCLK1 can autophosphorylate residues in its microtubule-binding doublecortin domain and in its autoinhibitory C-terminal domain, and phosphorylation of these residues can modulate the affinity with which DCLK1 binds microtubules [55]. Thus, it is possible that ZYG-8 autophosphorylates itself to regulate its own localization or activity. Future studies will be important to reveal precisely how kinase activity contributes to ZYG-8’s functions during mitosis and meiosis.

### Additional roles for ZYG-8 in spindle function

Importantly, our work also suggests that ZYG-8 may contribute to spindle organization and function in ways that do not involve kinase activity. We found that the *zyg-8*^*D604N*^ kinase dead mutant had elongated bipolar spindles but did not display some of the other defects observed in the *zyg-8* temperature sensitive mutants or following ZYG-8 depletion; *zyg-8*^*D604N*^ spindles largely had organized poles and were not significantly bent. Thus, ZYG-8 likely has both kinase-dependent and independent functions.

Doublecortin-family proteins have been shown to bind microtubules directly to regulate aspects of microtubule dynamics, including decreasing the catastrophe frequency and increasing the nucleation rate [32,33,56–60]. Since ZYG-8 localizes broadly throughout the spindle, it is possible that it directly modulates microtubule dynamics via its microtubule-binding doublecortin domain. Additionally, it is possible that ZYG-8 could impact motor activity via its microtubule-binding ability. In mammals, DCLK1 and its paralog DCX (doublecortin) are required for neuronal development and can regulate motor activity in neurons [28–32]. Neurons lacking DCX or DCLK1 show defects in kinesin-mediated vesicle transport in the absence of defects in microtubule organization [61,62]. Moreover, DCLK1 coats dendritic microtubules and is required for kinesin-3-mediated cargo transport into dendrites; this led to the hypothesis that crosstalk between DCLK1 and motors at the microtubule surface represented a regulatory mechanism for motor-driven cargo transport [63]. Thus, it is also possible that in *C*. *elegans* oocytes, microtubule-bound ZYG-8 may regulate the association of motors with the microtubule lattice, affecting their activity. Further experiments will be crucial to understand the various ways that ZYG-8 contributes to the regulation of microtubule dynamics and spindle forces.

In summary, our studies have provided new insights into the functions of a doublecortin-family protein in cell division. Moreover, our work has shed light on how forces are balanced within acentrosomal spindles, and on how the bipolar spindle structure is stabilized. Future studies aimed at elucidating the relationship between ZYG-8, motors, and microtubule regulatory proteins should be performed to better understand how they work together to create a stable bipolar spindle with properly balanced forces.

## Materials and methods

### Strains

All *C*. *elegans* strains used in this study are shown in Table 1.

**Table 1:** *C*. *elegans* strains used in this study.

Name	Description	Genotype
N2	Original Bristol isolate	Wild type
DH235	*zyg-8* temperature sensitive mutant (kinase domain)	*zyg-8(b235ts) III*
FM49	*zyg-8* temperature sensitive mutant (Doublecortin domain)	*zyg-8(or484ts) III*
PHX3347	ZYG-8 AID strain: GFP::degron::ZYG-8; TIR1::mRuby	*zyg-8(syb3347)[gfp*::*degron*::*zyg-8]*, *unc-119(ed3) III; ieSi38 [Psun-1*::*TIR1*::*mRuby*:: *sun-1 3’UTR + Cbr-unc-119(+)] IV*
SMW44	mCherry::histone; GFP::tubulin; TIR1::mRuby live imaging strain	*ltSi220[pOD1249/pSW077; Pmex-5*::*GFP-tbb-2-operon-linker-mCherry-his-11*, *cb-unc119(+)] I; ieSi38 [Psun-1*::*TIR1*::*mRuby*:: *sun-1 3’UTR + Cbr-unc-119(+)] IV*.
SV1005	*bmk-1* mutant	*bmk-1(ok391) V*
SMW57	ZYG-8 AID, mCherry::histone, GFP::tubulin live imaging strain	*ltSi220 [pOD1249/pSW077; Pmex-5*::*GFP-tbb-2-operon-linker-mCherry-his-11*, *cb-unc119(+)] I; ieSi38 [Psun-1*::*TIR1*::*mRuby*:: *sun-1 3’UTR + Cbr-unc-119(+)] IV; zyg-8(syb3347)[gfp*::*degron*::*zyg-8] III*
SMW70	ZYG-8 AID; *bmk-1* mutant strain	*zyg-8(syb3347)[gfp*::*degron*::*zyg-8]*, *unc-119(ed3) III; ieSi38 [Psun-1*::*TIR1*::*mRuby*:: *sun-1 3’UTR + Cbr-unc-119(+)] IV; bmk-1(ok391) V*
PHX7782	ZYG-8 kinase dead strain (D604N)	*zyg-8(syb7782) III/hT2[bli-4(e937)let-*?*(qIs48)](I;III)*
SMW74	ZYG-8 AID; *bmk-1* mutant, mCherry::histone, GFP::tubulin live imaging strain	*ltSi220[pOD1249/pSW077; Pmex-5*::*GFP-tbb-2-operon-linker-mCherry-his-11*, *cb-unc119(+)] I; ieSi38 [Psun-1*::*TIR1*::*mRuby*:: *sun-1 3’UTR + Cbr-unc-119(+)] IV; zyg-8(syb3347) [gfp*::*degron*::*zyg-8] III; bmk-1(ok391) V*

### Generation of *C*. *elegans* strains and maintenance

PHX3347 and PHX7782 were generated by SunyBiotech using CRISPR/Cas9 editing of the endogenous locus of *zyg-8*. All strains were maintained at 15°C. The *zyg-8*^*D604N*^ mutation was embryonic lethal when homozygous, so the strain was balanced with the hT2 balancer. Homozygotes were over 98% embryonic lethal. Note that we were unable to evaluate whether the D604N mutation affected the stability of ZYG-8 via either immunofluorescence or western blotting, since we do not have a ZYG-8 antibody (depletion of the ZYG-8 AID strain was assessed using an antibody to the GFP tag). Since this mutation did not cause a complete loss of function phenotype, we infer that the mutated protein is not completely unstable. However, it is still possible that this mutation could lead to a reduction in protein levels that could explain the partial loss of function phenotype.

### Immunofluorescence

Immunofluorescence was performed as previously described [64]. Briefly, adult worms were picked into a 10 μL drop of Meiosis Medium (0.5 mg/mL Inulin, 25 mM HEPES, and 20% FBS in Leibovitz’s L-15 Media) [65] on a poly-L-lysine-coated glass slide. Worms were dissected to release oocytes, a coverslip was placed on top, and then the slide was plunged into liquid nitrogen and the coverslip was flicked off. Slides were fixed in -20°C methanol for 30–40 minutes (40 minutes for strains expressing GFP or mCherry in order to quench the fluorescence), washed in PBS, and then blocked in AbDil (PBS with 4% BSA, 0.1% Triton-X-100, 0.02% sodium azide) at room temperature for at least 30 minutes. Primary antibodies were diluted in AbDil and incubated at 4°C overnight. The next day, samples were washed 3X with PBS-T (PBS with 0.1% Triton-X-100) and incubated in secondary antibody diluted in AbDil for 2 hours. Samples were again washed 3X with PBS-T and incubated in mouse anti-α-Tubulin-FITC (Sigma) diluted 1:500 in Abdil at room temperature for 1 hour. Samples were rinsed again and then incubated at room temperature in Hoechst diluted 1:1000 in PBS-T for 15 minutes. Samples were then rinsed one final time in PBS-T and were mounted in mounting media (0.5% p-phenylenediamine, 20 mM Tris-Cl, pH 8.8, 90% glycerol) then sealed with nail polish and stored at 4°C. Slides were imaged within 2 weeks of production. Primary antibodies used were rabbit-α-ASPM-1 (1:5000, gift from Arshad Desai), rabbit-α-BMK-1 (1:500, gift from Jill Schumacher), mouse-α-Tubulin-FITC (1:500, DM1α, Sigma), and mouse-α-GFP (1:250, 3E6, Invitrogen). Alexa Fluor secondary antibodies (Invitrogen) were used at 1:500. Fixed imaging was performed on DeltaVision Core deconvolution microscope with a 100X (NA = 1.4) objective (Applied Precision). This microscope is housed in the Northwestern University Biological Imaging Facility supported by the NU Office for Research. Image stacks were obtained at 0.2μm z-steps and deconvolved using SoftWoRx (Applied Precision).

### RNAi feeding

RNAi was performed as previously described [64]. Briefly, individual RNAi clones from an RNAi library [66], were grown in LB containing 100μg/ml AMP overnight at 37°C. After 18 hours, cultures were pelleted for 10 minutes, and excess LB was removed. The pellet was resuspended in the remaining LB and spotted on agar plates containing 100μg/ml AMP and 1mM IPTG. Plates were kept in the dark at room temperature to dry overnight. Worms were synchronized by bleaching adult worms, collecting embryos, and allowing them to hatch without food overnight. These starved L1s were then plated onto the RNAi plates and grown to adulthood at 15°C for 5 days.

### Auxin treatment

#### Long-term Auxin treatment

NGM plates were prepared with auxin added to a final concentration of 1mM. Plates were stored in the dark and used within 8 weeks. 18 hours prior to fixation, L4 worms were picked onto an auxin-containing plate and left at 15°C. For experiments requiring both RNAi and auxin treatment, NGM plates were made containing 100μg/ml AMP, 1mM IPTG, and 1mM auxin, the plates were seeded with RNAi clones, and RNAi was performed as described above.

#### Short-term Auxin treatment

Meiosis Medium containing 5mM auxin was prepared using a stock of 400mM auxin dissolved in 100% EtOH. Adult worms were picked into this auxin solution and kept in a humidity chamber at room temperature for 30–45 minutes. Worms were then dissected and immunofluorescence was performed. As a vehicle control, EtOH was diluted in Meiosis Medium and the same incubation conditions were used.

### Ex Utero Live Imaging

Two-color live imaging was performed using a Nikon SoRa spinning disk confocal microscope with an oil-immersion 60x (1.42 NA) objective lens. A Yokogawa CSU-W1 dual-disk spinning disk unit with 50μm pinholes and a Hamamatsu ORCA-Fusion Digital CMOS Camera were used for image acquisition. The microscope was controlled using Nikon imaging software NIS-Elements. The SoRa microscope is housed in the Northwestern University Biological Imaging Facility supported by the NU Office for Research.

10–15 worms were taken from desired experimental plates and dissected into 10μl of Meiosis Medium (described above) either with or without 500μM auxin. Quickly, a small Vaseline ring was made around the sample and an 18x18mm coverslip was laid on top. The slide was imaged immediately. Fifteen z-stacks at 1μm increments were taken every 20 seconds at room temperature. Since we have found that oocytes begin to arrest and eventually die after extended light exposure, we filmed for a maximum of 15 minutes and discarded any movies that exhibited signs of arrest (e.g., movies in which the chromosomes stopped moving before the end of the timecourse); to determine the effects of protein depletion beyond this 15 minute timeframe, we used short-term AID. Exposure times and laser power were the same for all movies. Images were processed using ImageJ. Images are shown as maximum intensity projections of the entire spindle structure. In some movies, the chromosome signal was artificially brightened when the movie was processed to make them easier to see, as the mCherry::histone signal was sometimes quite dim; the GFP signal was not modified in any way.

To observe GFP::tubulin in the ZYG-8 AID live imaging strain, the spindle was exposed to 15% laser power for 200ms. To determine whether these settings would also detect ZYG-8 (which is also tagged with GFP in the ZYG-8 AID strain), we imaged ZYG-8 AID oocytes in a strain that lacked GFP::tubulin (and instead had an mCherry::tubulin transgene to visualize the spindle). Under the same imaging conditions (15% laser power for 200ms), no GFP signal was detected on the spindle in this strain, demonstrating that GFP::ZYG-8 is undetectable with the live imaging settings we used. In order observe any GFP::ZYG-8 signal, the cell has to be exposed to 30% laser power for 400ms. Cells cannot be filmed for long periods of time at a laser power higher than 20%, or they begin to die. Therefore, in the acute ZYG-8 AID and FRAP experiments, the GFP signal is provided by GFP::tubulin and not by GFP::ZYG-8.

### Fluorescence Recovery After Photobleaching (FRAP)

FRAP experiments were performed using the same Nikon SoRa spinning disk confocal microscope with an oil-immersion 60x (1.42 NA) objective lens. Worms were mounted with the same set-up used in ex-utero live imaging. Photobleaching was performed using a 405nm laser. The midspindle was bleached with a 2.0 ferret ROI and poles were bleached with a 2.5 ferret ROI, both at 20% laser power with a 10μs dwell time. Each frame was exposed for 200ms with the delay between acquisitions. Image analysis was performed using Nikon SoRa imaging software NIS-Elements AR. If spindles moved during acquisition, the ROI was manually moved with spindle as the movie was acquired. Raw fluorescence intensity data were exported to an Excel file and then the values were normalized. The fluorescence intensity for the bleached region was normalized to 1.0 for the frame immediately before bleaching, while t = 0 (the frame immediately after bleaching) was normalized to 0 for the bleached region and to 1.0 for the unbleached region. Recovery curves and half-time recovery values were generated using a custom Python script (https://github.com/YuntongZou/FRAP-code).

### Western blotting

For long-term auxin treatment, adult worms were transferred to either four control NGM or four NGM auxin-containing plates (100 worms/plate) then incubated for 24 hours. These plates were then washed into an Eppendorf tube (4 plates/tube) and bleached to remove worm bodies and leave only embryos. Bleached samples were spun at 800rcf for 1 minute, rinsed with M9 and spun again, and then liquid was removed, leaving the bleached embryo sample in 10μl M9. For short-term auxin treatment, 4 NGM plates of approximately 100 adult worms each were washed into an Eppendorf tube with 500μl of Meiosis Medium containing either 5mM auxin or vehicle. The worms were incubated with intermittent tube rotation for 40 minutes without light exposure. After 40 minutes, worms were bleached as described above leaving 10μl of M9 and embryo sample. 10μl samples were then mixed with 10μl SDS sample buffer, boiled at 95°C for 10 minutes, and then stored at -20°C immediately.

Samples were run on 4–20% gradient Tris-Glycine gel (BioRad Mini-PROTEAN TGX) at 80V. The protein was wet transferred onto nitrocellulose at 120V for 22 hours. The blot was blocked in 5% milk with TBS-0.1%Tween for 2 hours at room temperature on a rocker and then incubated with the indicated primary antibodies overnight at 4°C (1:1000 mouse anti-degron (Sigma-Aldrich) or 1:5000 mouse anti-tubulin (ThermoFisher)). The blot was washed with TBS-0.1% Tween and incubated with goat anti-mouse HRP (1:5000) (ThermoFisher) for 2 hours at room temperature, washed once more, incubated for 5 minutes in BioRad Clarity ECL solution, and then exposed for 10 minutes.

### Data analysis and quantification

#### Western blot quantification

Raw intensity of the band signal was quantified using Fiji. A ROI was created around the control band signal and raw signal intensity was obtained. The same size ROI was used to obtain raw signal intensity from short-term and long-term bands, and from a background region. Background was then subtracted from each raw intensity value.

#### Meiosis I vs. Meiosis II spindles

Meiotic stage was determined by counting chromosomes and assessing their size. In wild-type oocytes, MI spindles have 6 bivalents and MII spindles have 6 chromosomes, but MII chromosomes can be distinguished from bivalents because they are smaller. A failure in polar body extrusion in MI would result in extra chromosomes in MII.

#### Spindle length

To determine spindle length, three-dimensional renderings of spindles were generated using Imaris 3D Imaging Software (Bitplane). The center point of each pole was determined using the Surfaces tool to define the volume of the ASPM-1 staining, and the center point of this volume was assigned. Length was then measured as the center to center of each pole.

#### Spindle angle

The angle of the spindle was measured using Imaris Software. The poles were defined as described above. To determine the midspindle, the Surfaces tool was used to define the volume of the DNA signal and the center point of this volume was assigned; this was then used as the vertex while the poles were used as the arms, and the inside angle of the spindle was measured.

#### Number of poles

Quantifications were performed by counting poles per spindle as defined by ASPM-1 staining. A pole was defined as ASPM-1 staining at the end of MT bundles, with an accumulation of more than 3 ASPM-1 foci.

#### Number of ASPM-1 foci at spindle periphery

Quantifications were performed by counting the number of ASPM-1 foci at the end of MT bundles at the periphery of monopolar spindles.

#### Mitotic spindle positioning

Mitotic spindle positioning was determined using 2D whole cell images. The center of each spindle was determined using the Surfaces tool to define the DNA signal and find its center point. The cell center was determined by measuring the length of the embryo along the long axis and the width of the embryo along the short axis, and setting the center as the halfway point of the total length and width of the embryo. Distance from spindle center to cell center was measured.

### Statistical methods

Statistical analysis was performed using R studio; a Shapiro-Wilks test was used to confirm data distribution normality. Normally distributed data was analyzed using a two-tailed t-test, while non-normally distributed data was analyzed using a Mann-Whitney Wilcoxon non-parametric test. Comparison of spindle angle data was performed using a one-way ANOVA.

## Supporting information

S1 Fig A *zyg-8* temperature sensitive mutant displays defects in oocyte spindle morphology at the restrictive temperature. (A) ZYG-8 schematic, highlighting the microtubule binding doublecortin domain, the kinase domain, and the location of the *b235* and *or484* temperature sensitive mutations. (B) Immunofluorescence images of *zyg-8(b235)* oocytes at either the permissive (15°C) or restrictive (25°C) temperatures. Shown are tubulin (green), DNA (blue), and ASPM-1 (red). (C-E) Quantification of spindle length, spindle angle, and number of ASPM-1-marked poles for the experiment shown in B. After incubation at the restrictive temperature, oocyte spindles were on average longer (p<0.001), more bent (p<0.05), and some had additional ASPM-1-marked poles. (F-G) Comparison of the temperature sensitive strains at the permissive and restrictive temperatures to wild-type (N2) worms. Spindles in each temperature sensitive mutant at the restrictive temperature were significantly longer than control (N2) spindles (p<0.001) but were not significantly longer at the permissive temperature (p>0.1). Similarly, spindles in each mutant were more bent than control spindles at the restrictive temperature (*or484ts*: p<0.001; *b235ts*: p<0.01), but not at the permissive temperature (p>0.1). Scale bars = 5μm.
(TIF)

S2 Fig AID allows for temporally and spatially controlled depletion of ZYG-8. (A) Western blot of control, short-term auxin treated (soaking worms for 40 minutes in auxin-containing media), and long-term auxin treated (incubating worms for 18 hours on auxin-containing plates) samples. An anti-degron antibody was used to detect ZYG-8 and an anti-tubulin antibody was used as a loading control. Quantification of band intensity revealed substantial depletion of ZYG-8 following both short-term AID (~63% reduction) and long-term AID (~93%). Note that we are unable to quantify the extent of ZYG-8 depletion following acute AID; this protocol relies on dissecting oocytes into auxin, and it would be technically difficult to collect enough of these oocytes to generate a gel sample for western blot analysis. However, the strong spindle phenotypes we observe via live imaging of acute ZYG-8 AID suggest that we are also achieving strong depletion using this method. (B) Immunofluorescence images of one-cell stage mitotically dividing embryos in the ZYG-8 AID strain. Auxin treatment resulted in spindle positioning defects, phenocopying previous studies of *zyg-8* mutants [22, 23]. Quantification reflects the distance measured from the spindle center to the cell center (p<0.005). (C) Quantification of spindle angle and spindle length in the ZYG-8 AID strain compared to a control strain expressing TIR1 without ZYG-8 tagged; the lengths and angles did not appear significantly different (p>0.1), suggesting that tagging ZYG-8 does not substantially alter protein function. Scale bar = 10μm.
(TIF)

S3 Fig Short-term ZYG-8 depletion in unarrested oocytes reveals the same phenotypes observed following metaphase-arrest. (A) Immunofluorescence images of unarrested oocytes treated with vehicle (row 1) or short-term auxin (rows 2–3); shown are tubulin (green), DNA (blue), and ASPM-1 (red). (B-D) Quantification of the number of ASPM-1-marked poles, spindle angle, and spindle length. Short term ZYG-8 AID results in spindles that are longer (p<0.001) and more angled (p<0.005) even without metaphase arrest. In panel D, the data for long-term AID and short-term *emb-30(RNAi)* AID are repeated from Figs 2F and 3C, so that the three conditions can be easily compared. (E) Live imaging of acute auxin treatment of unarrested spindles; shown are GFP::tubulin (green) and mCherry::histone (magenta). Control spindles maintain bipolarity and eventually segregate chromosomes in anaphase (rows 1–2). In contrast, rows 3–4 show an auxin-treated unarrested spindle elongate and weaken at the midspindle, demonstrating the same defects observed with metaphase arrest. Scale bars = 5μm.
(TIF)

S4 Fig ZYG-8 depletion causes spindle phenotypes in a *bmk-1* mutant without KLP-18 depletion. (A) Immunofluorescence images of oocyte spindles in the ZYG-8 AID, *bmk-1(ok391)* strain in the presence and absence of auxin. Due to diffuse ZYG-8 localization, ZYG-8 images are not deconvolved. (B-C) Quantification of spindle length and the number of ASPM-1-marked poles per spindle. Spindles are longer upon auxin depletion (p<0.001) and a fraction of spindles have multiple poles, demonstrating that ZYG-8 has functions in addition to regulating BMK-1. Scale bars = 5μm.
(TIF)

S5 Fig Exposure to auxin does not affect microtubule turnover when ZYG-8 is not degron-tagged. Fluorescence recovery after photobleaching (FRAP) was performed on oocytes expressing TIR1 and GFP::tubulin. (A) Stills from FRAP movies; 3 frames were acquired before bleaching and then bleaching occurred at t = 0. The laser was focused near the spindle pole. The white dotted circles denote the ROIs within the bleached region where fluorescence intensity was measured. (B) Graphs showing GFP::tubulin intensity throughout the FRAP timecourse. Bleached ROIs are represented with the green traces, and the reference (unbleached) pole is represented with the orange traces. The solid lines are the average, and the standard error of the mean is shaded. t½ was calculated from fitting the recovery curve to a single exponential function. Auxin treatment does not appreciably impact tubulin turnover when ZYG-8 is not degron-tagged. Scale bar = 5μm.
(TIF)

S1 Video ZYG-8 AID metaphase-arrested oocyte spindles maintain bipolarity in the absence of auxin. Live imaging of an *emb-30(RNAi)* metaphase-arrested oocyte spindle; corresponds to Fig 3A. Shown are GFP::tubulin (green) and mCherry::histone (magenta). Oocytes were dissected into control Meiosis Medium containing vehicle. The spindle maintains bipolarity; chromosomes oscillate but stay aligned at the midspindle. The phenotype was consistent in all videos (n = 5). Scale bar = 5μm.
(MOV)

S2 Video ZYG-8 AID metaphase-arrested oocyte spindles elongate and lose midspindle integrity upon auxin treatment. Live imaging of an *emb-30(RNAi)* metaphase-arrested oocyte spindle; corresponds to Fig 3A. Shown are GFP::tubulin (green) and mCherry::histone (magenta); the brightness of the chromosomes was increased to make them easier to see. Oocytes were dissected into auxin-containing Meiosis Medium. The spindle immediately begins to elongate, the midspindle weakens, and chromosomes lose alignment at the midspindle. The phenotype was consistent in all videos (n = 10). Scale bar = 5μm.
(MOV)

S3 Video ZYG-8 AID unarrested oocyte spindles maintain bipolarity and undergo anaphase in the absence of auxin. Live imaging of a control oocyte spindle; corresponds to S3E Fig. Shown are GFP::tubulin (green) and mCherry::histone (magenta). Oocytes were dissected into control Meiosis Medium containing vehicle. The spindle maintains bipolarity in metaphase, rotates towards the cortex, shortens, and then elongates as chromosomes segregate bidirectionally. The phenotype was consistent in all videos (n = 5). Scale bar = 5μm.
(MOV)

S4 Video ZYG-8 AID unarrested oocytes exhibit spindle defects following auxin treatment. Live imaging of an unarrested auxin-treated oocyte spindle; corresponds to S3E Fig. Shown are GFP::tubulin (green) and mCherry::histone (magenta). Oocytes were dissected into auxin-containing Meiosis Medium. The auxin-treated spindle begins to elongate as the midspindle loses integrity, and chromosome become misaligned. Spindle defects were observed in all videos (n = 5). Scale bar = 5μm.
(MOV)

S5 Video *klp-18(RNAi)* spindles maintain a single monopole as chromosomes move towards the center of the aster during anaphase. Live imaging of a *klp-18(RNAi)* ZYG-8 AID oocyte spindle; corresponds to Fig 5C. Shown are GFP::tubulin (green) and mCherry::histone (magenta); the brightness of the chromosomes was increased to make them easier to see. Oocytes were dissected into control Meiosis Medium containing vehicle. Control *klp-18(RNAi)* spindles remain monopolar as chromosomes slowly move towards the center pole in anaphase. The phenotype was consistent in all videos (n = 5). Scale bar = 5μm.
(MOV)

S6 Video Acute ZYG-8 AID causes monopolar spindles to reorganize, reestablish bipolarity, and segregate chromosomes bidirectionally, example 1. Live imaging of a *klp-18(RNAi)* ZYG-8 AID oocyte spindle; corresponds to Fig 5C. Shown are GFP::tubulin (green) and mCherry::histone (magenta); the brightness of the chromosomes was increased to make them easier to see. Oocytes were dissected into auxin-containing Meiosis Medium. Upon treatment with auxin, the monopolar spindle reorganizes into a bipolar spindle that then segregates chromosomes bidirectionally (bidirectional chromosome segregation was observed in 4/12 oocyte spindles). Scale bar = 5μm.
(MOV)

S7 Video Acute ZYG-8 AID causes monopolar spindles to reorganize and reestablish bipolarity, example 2. Live imaging of a *klp-18(RNAi)* ZYG-8 AID Meiosis II oocyte spindle; corresponds to Fig 5C. Shown are GFP::tubulin (green) and mCherry::histone (magenta). Oocytes were dissected into auxin-containing Meiosis Medium. Upon treatment with auxin, the monopolar spindle reorganizes and incorporates the polar body, forming a multipolar and finally a bipolar spindle. Monopolar spindles reorganized in 12/12 videos: 3/12 reincorporated the polar body, 6/12 reestablished bipolarity, and 3/12 formed disorganized spindles. Scale bar = 5μm.
(MOV)

S8 Video ZYG-8 AID; *bmk-1(ok391)* monopolar spindles maintain a single pole and chromosomes move inwards in anaphase in the absence of auxin. Live imaging of a *klp-18(RNAi)* ZYG-8 AID *bmk-1(ok391)* oocyte spindle; corresponds to Fig 6C. Shown are GFP::tubulin (green) and mCherry::histone (magenta). Oocytes were dissected into control Meiosis Medium containing vehicle. The monopolar spindle maintains a single pole as chromosomes move towards the center of the spindle in anaphase. The phenotype was consistent in all videos (n = 5). Scale bar = 5μm.
(MOV)

S9 Video ZYG-8 AID; *bmk-1(ok391)* monopolar spindles maintain a single pole and chromosomes move inwards in anaphase following auxin treatment. Live imaging of a *klp-18(RNAi)* ZYG-8 AID *bmk-1(ok391)* oocyte spindle; corresponds to Fig 6C. Shown are GFP::tubulin (green) and mCherry::histone (magenta). Oocytes were dissected into auxin-containing Meiosis Medium. After auxin treatment, the monopolar spindle maintains a single pole as chromosomes move towards the center of the spindle in anaphase. The phenotype was consistent in all videos (n = 5). Scale bar = 5μm.
(MOV)

S10 Video FRAP of the pole region in the ZYG-8 AID strain without auxin. Live imaging of a ZYG-8 AID oocyte spindle in the absence of auxin; corresponds to Fig 7A. GFP::tubulin is shown in green. Oocytes were dissected into Meiosis Medium and filmed immediately. Three frames were acquired prior to bleaching. Time 0 represents bleaching. The phenotype was consistent in all videos (n = 8). Scale bar = 5μm.
(MOV)

S11 Video FRAP of the pole region following long-term ZYG-8 AID. Live imaging of a ZYG-8 AID oocyte spindle; corresponds to Fig 7A. GFP::tubulin is shown in green. Worms were incubated on auxin-containing plates for 18 hours prior to imaging. Oocytes were then dissected into Meiosis Medium and filmed immediately. Three frames were acquired prior to bleaching. Time 0 represents bleaching. The phenotype was consistent in all videos (n = 18). Scale bar = 5μm.
(MOV)

S12 Video FRAP of the midspindle in the ZYG-8 AID strain without auxin. Live imaging of a ZYG-8 AID oocyte spindle in the absence of auxin; corresponds to Fig 7C. GFP::tubulin is shown in green. Oocytes were dissected into Meiosis Medium and filmed immediately. Three frames were acquired prior to bleaching. Time 0 represents bleaching. The phenotype was consistent in all videos (n = 10). Scale bar = 5μm.
(MOV)

S13 Video FRAP of the midspindle following long-term ZYG-8 AID. Live imaging of a ZYG-8 AID oocyte spindle; corresponds to Fig 7C. GFP::tubulin is shown in green. Worms were incubated on auxin-containing plates for 18 hours prior to imaging. Oocytes were then dissected into Meiosis Medium and filmed immediately. Three frames were acquired prior to bleaching. Time 0 represents bleaching. The phenotype was consistent in all videos (n = 16). Scale bar = 5μm.
(MOV)

S1 Data Czajkowski_FRAP_data file. This file contains data for all of the FRAP experiments reported in this manuscript. The file is organized with multiple tabs, one tab for each of the traces shown in the graphs. Tabs labeled “reference” represent the unbleached regions, and tabs labeled “bleach” represent the photobleached regions. Each column within each tab represents the data for one individual movie.
(XLSX)

S2 Data Czajkowski_Length_and_angle_data file. This file contains the raw data for each of the spindle length and spindle angle graphs in the manuscript. The file is organized into multiple tabs, with each tab containing the data from one figure panel.
(XLSX)
