# Supplementary figures and images for: The doublecortin-family kinase ZYG-8DCLK1 regulates microtubule dynamics and motor-driven forces to promote the stability of C. elegans acentrosomal spindles
Source: PLoS Genet. 2024 Sep 3;20(9):e1011373. doi: 10.1371/journal.pgen.1011373 (PMC11398696; doi:10.1371/journal.pgen.1011373)

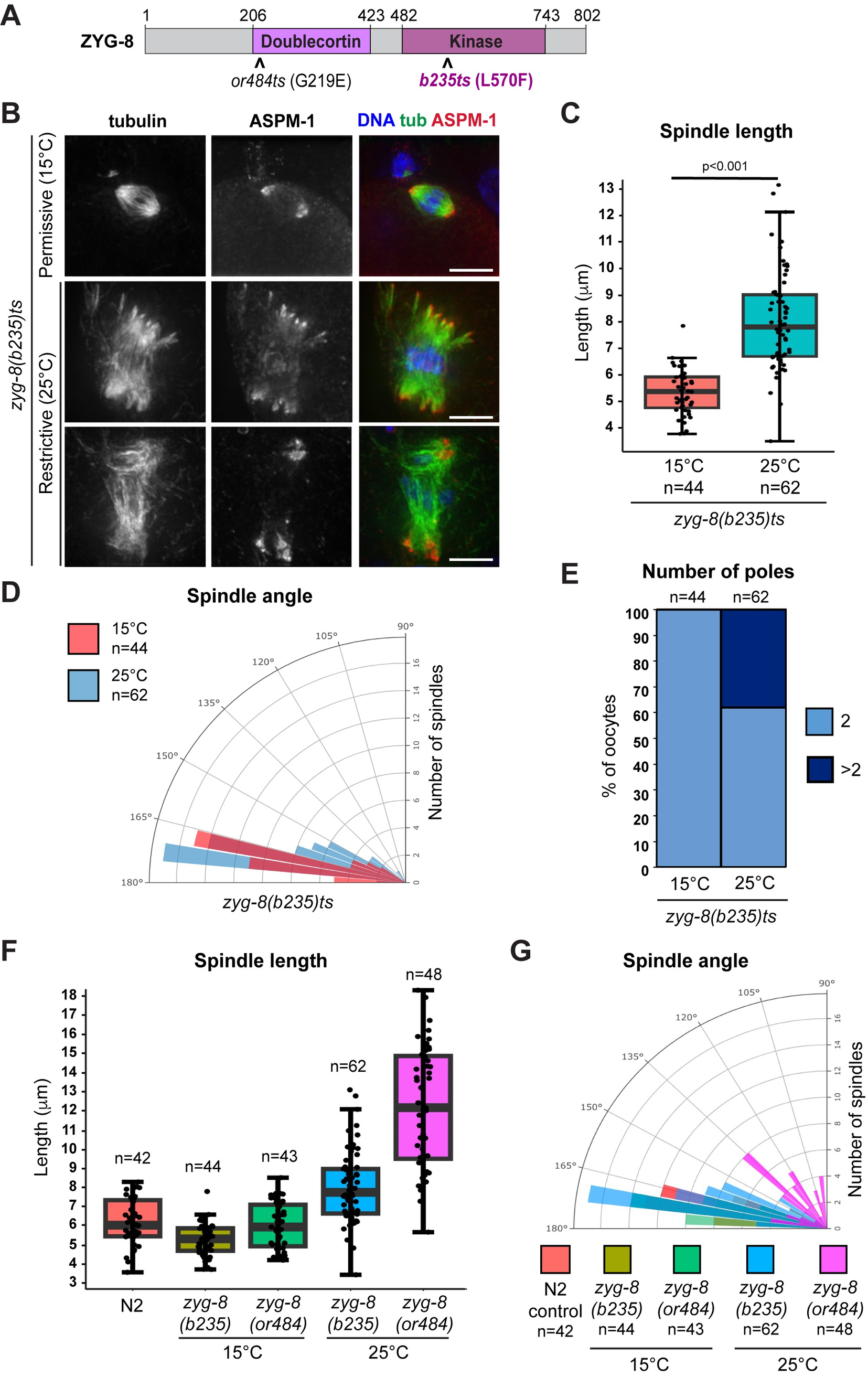

Supplement: S1 Fig — (A) ZYG-8 schematic, highlighting the microtubule binding doublecortin domain, the kinase domain, and the location of the b235 and or484 temperature sensitive mutations. (B) Immunofluorescence images of zyg-8(b235) oocytes at either the permissive (15°C) or restrictive (25°C) temperatures. Shown are tubulin (green), DNA (blue), and ASPM-1 (red). (C-E) Quantification of spindle length, spindle angle, and number of ASPM-1-marked poles for the experiment shown in B. After incubation at the restrictive temperature, oocyte spindles were on average longer (p<0.001), more bent (p<0.05), and some had additional ASPM-1-marked poles. (F-G) Comparison of the temperature sensitive strains at the permissive and restrictive temperatures to wild-type (N2) worms. Spindles in each temperature sensitive mutant at the restrictive temperature were significantly longer than control (N2) spindles (p<0.001) but were not significantly longer at the permissive temperature (p>0.1). Similarly, spindles in each mutant were more bent than control spindles at the restrictive temperature (or484ts: p<0.001; b235ts: p<0.01), but not at the permissive temperature (p>0.1). Scale bars = 5μm. (TIF) [file pgen.1011373.s001.tif]

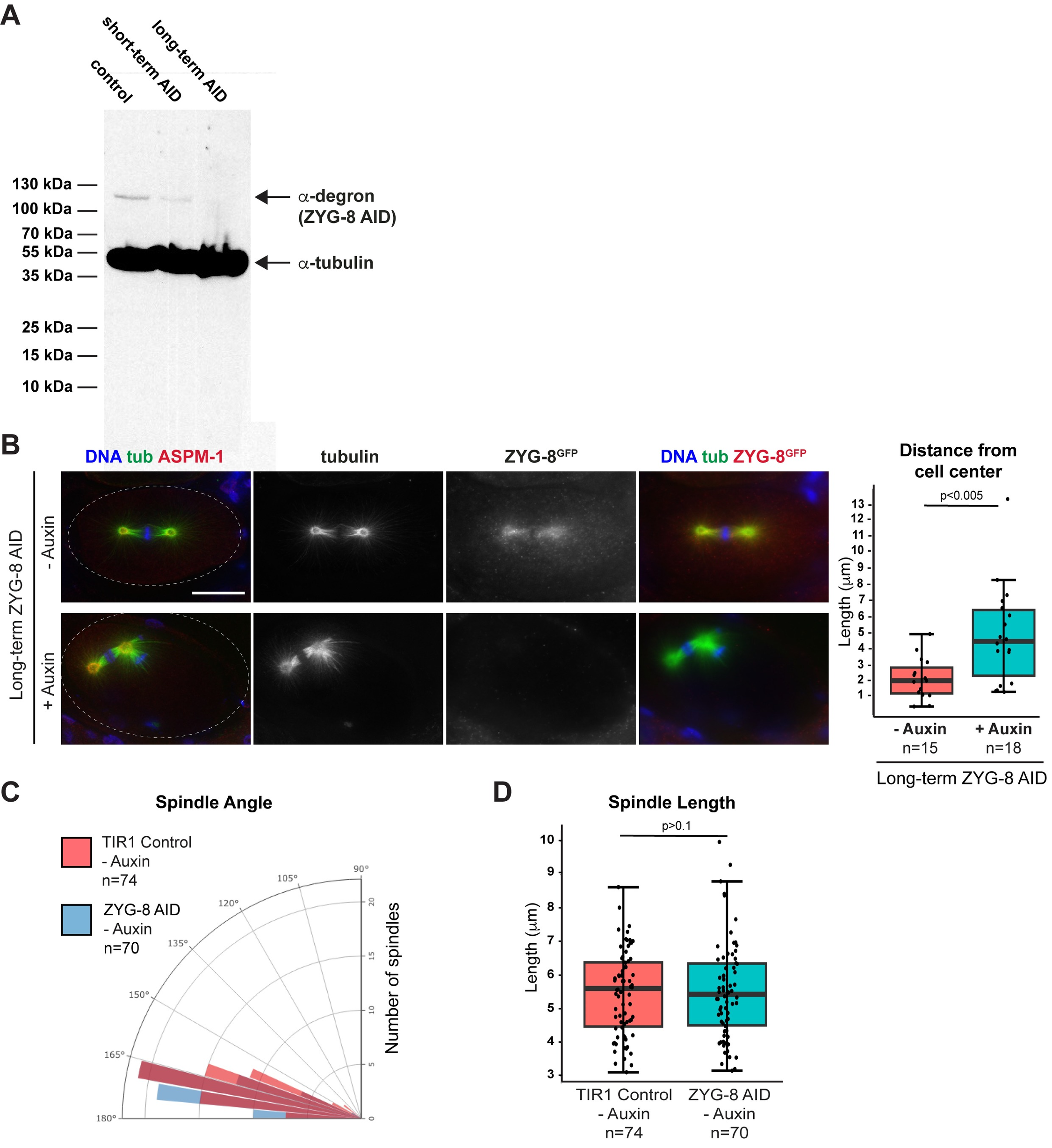

Supplement: S2 Fig — (A) Western blot of control, short-term auxin treated (soaking worms for 40 minutes in auxin-containing media), and long-term auxin treated (incubating worms for 18 hours on auxin-containing plates) samples. An anti-degron antibody was used to detect ZYG-8 and an anti-tubulin antibody was used as a loading control. Quantification of band intensity revealed substantial depletion of ZYG-8 following both short-term AID (~63% reduction) and long-term AID (~93%). Note that we are unable to quantify the extent of ZYG-8 depletion following acute AID; this protocol relies on dissecting oocytes into auxin, and it would be technically difficult to collect enough of these oocytes to generate a gel sample for western blot analysis. However, the strong spindle phenotypes we observe via live imaging of acute ZYG-8 AID suggest that we are also achieving strong depletion using this method. (B) Immunofluorescence images of one-cell stage mitotically dividing embryos in the ZYG-8 AID strain. Auxin treatment resulted in spindle positioning defects, phenocopying previous studies of zyg-8 mutants [22, 23]. Quantification reflects the distance measured from the spindle center to the cell center (p<0.005). (C) Quantification of spindle angle and spindle length in the ZYG-8 AID strain compared to a control strain expressing TIR1 without ZYG-8 tagged; the lengths and angles did not appear significantly different (p>0.1), suggesting that tagging ZYG-8 does not substantially alter protein function. Scale bar = 10μm. (TIF) [file pgen.1011373.s002.tif]

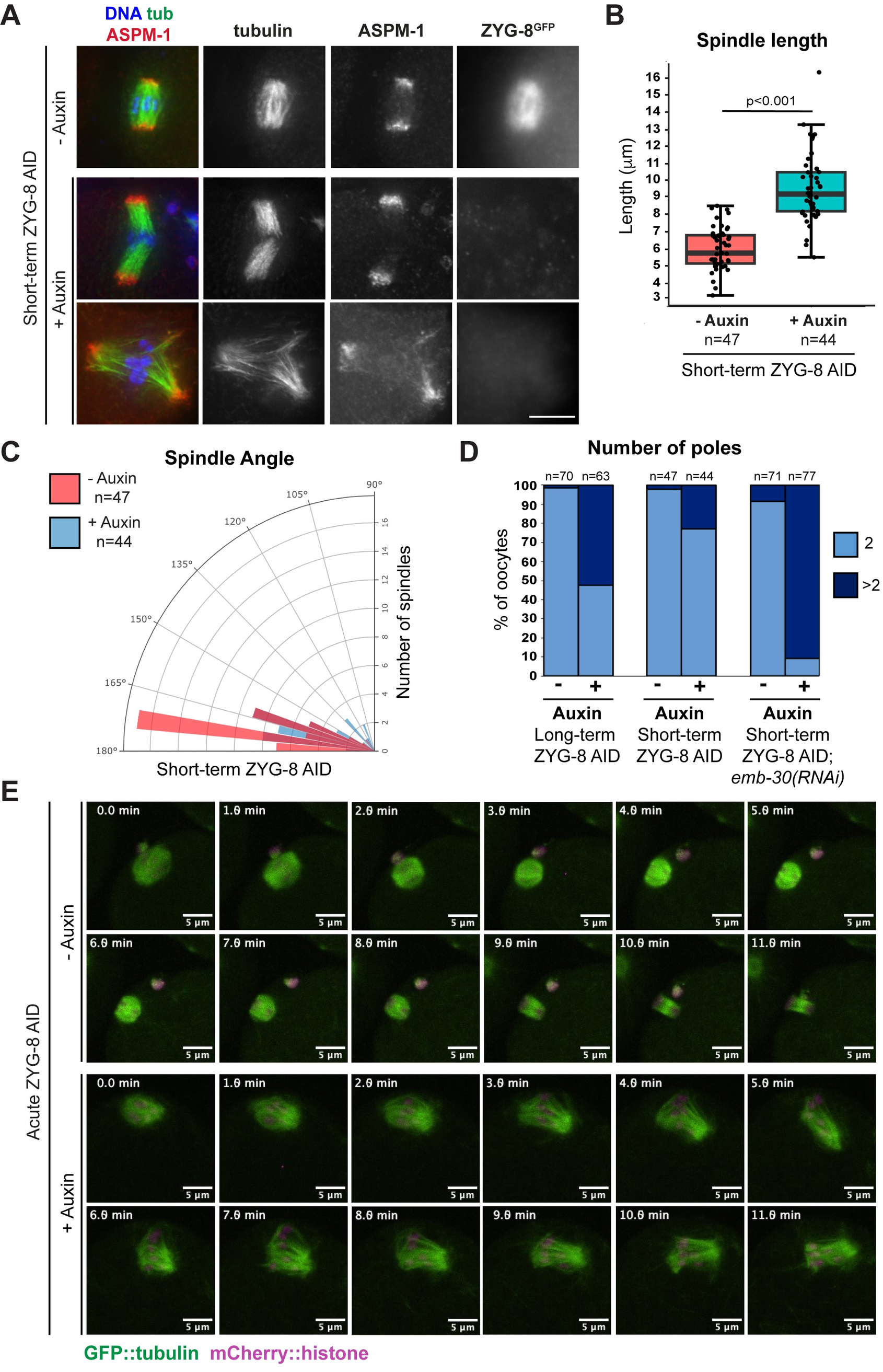

Supplement: S3 Fig — (A) Immunofluorescence images of unarrested oocytes treated with vehicle (row 1) or short-term auxin (rows 2–3); shown are tubulin (green), DNA (blue), and ASPM-1 (red). (B-D) Quantification of the number of ASPM-1-marked poles, spindle angle, and spindle length. Short term ZYG-8 AID results in spindles that are longer (p<0.001) and more angled (p<0.005) even without metaphase arrest. In panel D, the data for long-term AID and short-term emb-30(RNAi) AID are repeated from Figs 2F and 3C, so that the three conditions can be easily compared. (E) Live imaging of acute auxin treatment of unarrested spindles; shown are GFP::tubulin (green) and mCherry::histone (magenta). Control spindles maintain bipolarity and eventually segregate chromosomes in anaphase (rows 1–2). In contrast, rows 3–4 show an auxin-treated unarrested spindle elongate and weaken at the midspindle, demonstrating the same defects observed with metaphase arrest. Scale bars = 5μm. (TIF) [file pgen.1011373.s003.tif]

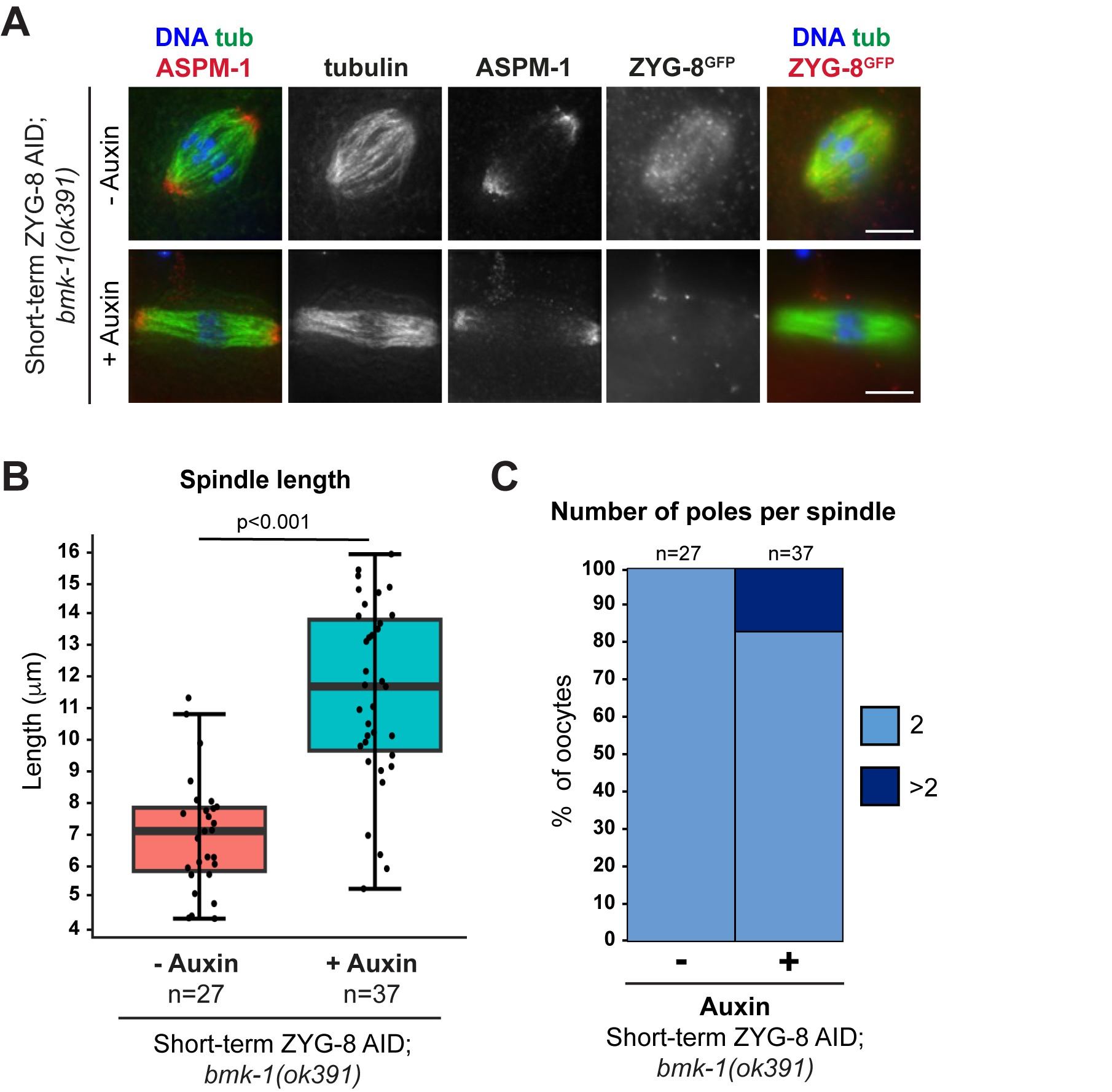

Supplement: S4 Fig — (A) Immunofluorescence images of oocyte spindles in the ZYG-8 AID, bmk-1(ok391) strain in the presence and absence of auxin. Due to diffuse ZYG-8 localization, ZYG-8 images are not deconvolved. (B-C) Quantification of spindle length and the number of ASPM-1-marked poles per spindle. Spindles are longer upon auxin depletion (p<0.001) and a fraction of spindles have multiple poles, demonstrating that ZYG-8 has functions in addition to regulating BMK-1. Scale bars = 5μm. (TIF) [file pgen.1011373.s004.tif]

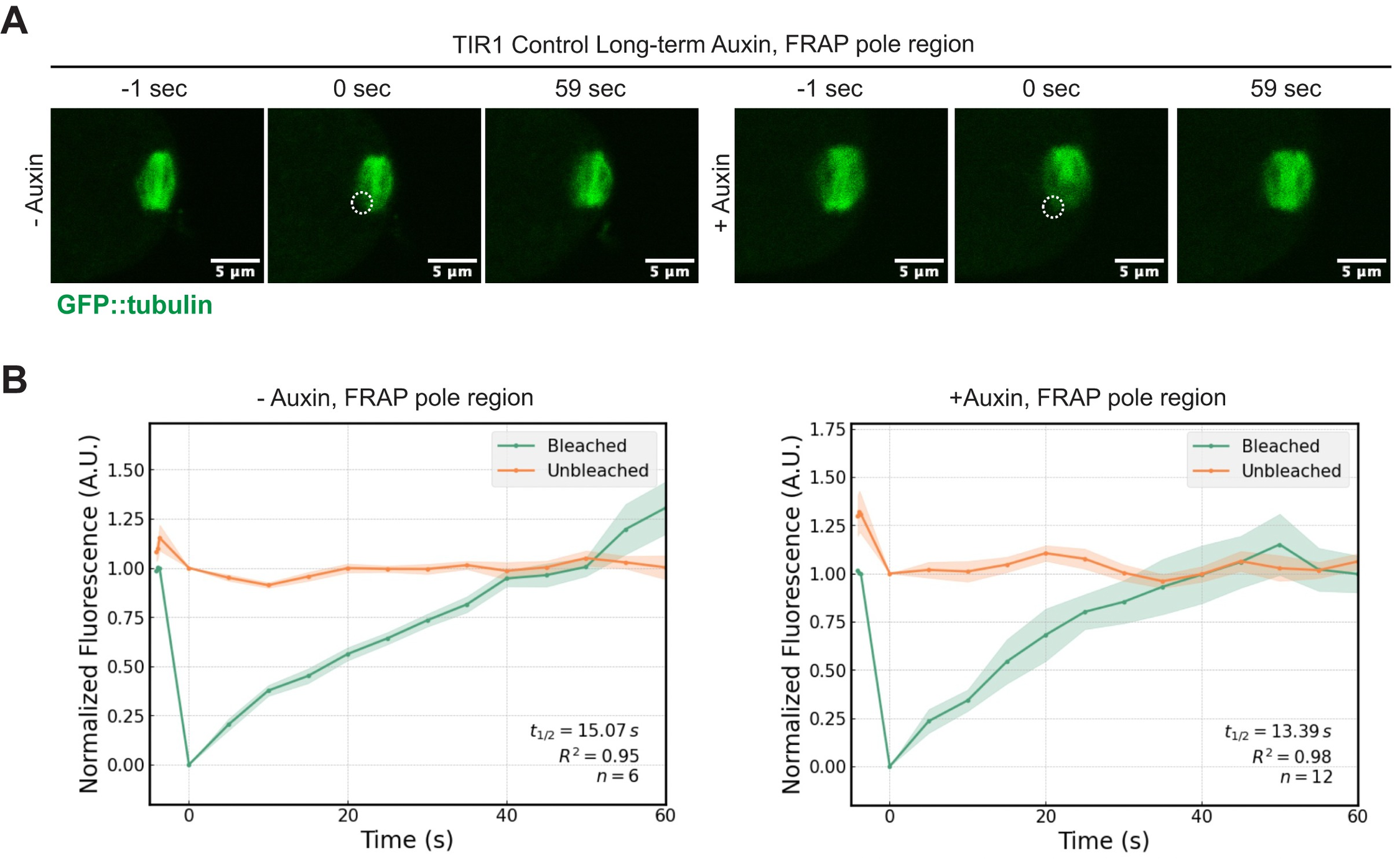

Supplement: S5 Fig — Fluorescence recovery after photobleaching (FRAP) was performed on oocytes expressing TIR1 and GFP::tubulin. (A) Stills from FRAP movies; 3 frames were acquired before bleaching and then bleaching occurred at t = 0. The laser was focused near the spindle pole. The white dotted circles denote the ROIs within the bleached region where fluorescence intensity was measured. (B) Graphs showing GFP::tubulin intensity throughout the FRAP timecourse. Bleached ROIs are represented with the green traces, and the reference (unbleached) pole is represented with the orange traces. The solid lines are the average, and the standard error of the mean is shaded. t½ was calculated from fitting the recovery curve to a single exponential function. Auxin treatment does not appreciably impact tubulin turnover when ZYG-8 is not degron-tagged. Scale bar = 5μm. (TIF) [file pgen.1011373.s005.tif]
